# Serum Total Cholinesterase Activity on Admission Is Associated with Disease Severity and Outcome in Patients with Traumatic Brain Injury

**DOI:** 10.1371/journal.pone.0129082

**Published:** 2015-06-24

**Authors:** Qing-Hong Zhang, An-Min Li, Sai-Lin He, Xu-Dong Yao, Jing Zhu, Zhi-Wen Zhang, Zhi-Yong Sheng, Yong-Ming Yao

**Affiliations:** 1 Key Research Laboratory of Tissue Repair and Regeneration of PLA, and Beijing Key Research Laboratory of Skin Injury, Repair and Regeneration; First Hospital Affiliated to the Chinese PLA General Hospital, Beijing, 100048, P. R. China; 2 Department of Neurosurgery, Hainan Branch of the Chinese PLA General Hospital, Sanya, Hainan, 572013, P. R. China; 3 Department of Neurosurgery, First Hospital Affiliated to the Chinese PLA General Hospital, Beijing, 100048, P. R. China; 4 Department of Emergency, First Hospital Affiliated to Wenzhou Medical University, Wenzhou, 325000, P. R. China; 5 Department of Laboratory Medicine, First Hospital Affiliated to the Chinese PLA General Hospital, Beijing, 100048, P. R. China; East Carolina University, UNITED STATES

## Abstract

**Background:**

Traumatic brain injury (TBI) is one of the leading causes of neurological disability. In this retrospective study, serum total cholinesterase (ChE) activities were analyzed in 188 patients for diagnostic as well as predictive values for mortality.

**Methods and Findings:**

Within 72 hours after injury, serum ChE activities including both acetylcholinesterase and butyrylcholinesterase were measured. Disease severity was evaluated with Acute Physiology and Chronic Health Evaluation (APACHE) II score, Glasgow Coma Score, length of coma, post-traumatic amnesia and injury feature. Neurocognitive and functional scores were assessed using clinical records. Of 188 patients, 146 (77.7%) survived and 42 (22.3%) died within 90 days. Lower ChE activities were noted in the non-survivors *vs*. survivors (5.94±2.19 *vs*. 7.04±2.16 kU/L, p=0.023), in septic *vs*. non-infected patients (5.93±1.89 *vs*. 7.31±2.45 kU/L, p=0.0005) and in patients with extremely severe injury *vs*. mild injury (6.3±1.98 vs. 7.57±2.48 kU/L, p=0.049). The trajectories of serum ChE levels were also different between non-survivors and survivors, septic and non-infected patients, mild and severely injured patients, respectively. Admission ChE activities were closely correlated with blood cell counts, neurocognitive and functional scores both on admission and at discharge. Receiver operating characteristic analysis showed that the area under the curve for ChE was inferior to that for either APACHE II or white blood cell (WBC) count. However, at the optimal cutoff value of 5 kU/L, the sensitivity of ChE for correct prediction of 90-day mortality was 65.5% and the specificity was 86.4%. Kaplan-Meier analysis showed that lower ChE activity (<5 kU/L) was more closely correlated with poor survival than higher ChE activity (>5 kU/L) (p=0.04). After adjusting for other variables, ChE was identified as a borderline independent predictor for mortality as analyzed by Binary logistic regression (P=0.078).

**Conclusions:**

Lowered ChE activity measured on admission appears to be associated with disease severity and outcome for TBI patients.

## Introduction

Traumatic brain injury (TBI) affects up to 10 million people globally [1], yet our ability to diagnose and treat TBI is deficient. Therefore, the identification of diagnostic and prognostic biomarkers that directly reflect injury to central nervous system (CNS) is imperative [2,3]. Stress, as in TBI, can be defined as a psychological, environmental, or physiologic threat on homeostasis [4], which largely involves the sympathetic and parasympathetic nervous systems. Catecholamine released from sympathetic nerve fibers launches a fight-or-flight reaction while eliciting both inflammatory and immunosuppressive responses [5]. The acetylcholine (ACh), a parasympathetic neurotransmitter, is another key contributor to stress to enhance neuronal excitability [6]. Aside from the vagus nerve, ACh could also be produced by peripheral leukocytes [7,8]. Then it can potently modulate classical immune response by activating α7 nicotinic acetylcholine receptor (α7 nAChR) on the leukocytes [9,10] in terms of “cholinergic anti-inflammatory response” [11].

Increasing evidence suggested that cholinergic status might be used to judge the disease severity and predict the potential risk of mortality [12]. Imbalanced sympathetic/parasympathetic activity was found to be associated with poor cardiovascular prognosis [13], worse stroke outcome [14], organ death in hypertension [15], and the sudden death as well as all-cause mortality [16]. Considering that ACh is extremely labile and difficult to use for clinical measurements [17], the employment of its hydrolyzing enzymes as an indirect measurement for parasympathetic dysfunction might be more suitable for clinical use. Cholinesterases (ChE), acetylcholinesterase (AChE), and the closely related enzyme butyrylcholinesterase (BChE), may contribute to the cholinergic status and parasympathetic dysfunction. AChE is the major cholinesterase in the brain in contrast to BChE, the major ACh-hydrolyzing enzyme in the circulation, both of which could mitigate the cholinergic anti-inflammatory pathway [18].

Although the morbidity and mortality of TBI are frequently attributed by the neurological consequences of the brain injury [19,20], non-neurological complications including cardiovascular, respiratory, and infection [21–23] could also affect its outcome. In fact, non-neurological consequences of brain injury show impressive similarities regardless of the type of brain insults, and appear to depend on the altered neuroimmune circuits [24], making patients more vulnerable to infection [25,26]. It has been documented that activation of the sympathetic nervous system (SNS) is crucial in CNS-induced immunodepression and inflammation, whereas depressed vagus activity may aggravate the inflammatory response [24]. Thus, cholinergic status as represented by ChE activity might be a potential biomarker for parasympathetic dysfunction in TBI.

So far, cholinergic status has been investigated mostly in brain tissue in TBI. Acutely elevated AChE activity was reported in the brain of ischemic [27] or blast injury [28], however, lowered AChE activity in the neocortex was seen in TBI patients with chronic cognitive symptoms [29]. For the practical purpose, the measurement of cholinergic status other than brain tissue is imperative. It was noted that serum ChE activity might be used in the diagnosis of stroke [14] and infection [30,31]. Although a significant decrease in serum ChE levels had been reported in human acute head injury [32], data regarding the association of ChE activity with blood cell counts, cognition, and neurofunctional outcome in TBI is extremely limited. Although it has not been verified yet, it is likely that increased vagal tone may account for the immune paralysis in TBI patients [33], in turn being prone to septic complications. Therefore, we assume that serum AChE activity would reflect the intensity of the cholinergic anti-inflammatory response toward brain damage and may be associated with TBI outcome. In the present study, we investigated the cholinergic predictor of the risk for the morbidity and mortality of TBI patients in two hospitals.

## Materials and Methods

### Patient inclusion and demographic data

Using a database of patients admitted to the Departments of Neurosurgery in two hospitals Affiliated to the Chinese PLA General Hospital (Beijing, China; Sanya, Hainan, China) between August 2009 and September 2013, we retrieved data on patients who were aged >15 years. Patients with leukemia, acute meningitis, hepatic disorder, cerebral vasculitis, or other recent CNS infection were excluded. The Institutional Review Board of Chinese PLA General Hospital approved the clinical study. Because the serum ChE activity was included in the panel examination monitoring the hepatic function, every patient with traumatic injury was tested for ChE activity on admission and frequently during hospital stay. Since the patients received no extra treatment or examination, the written informed consent from the patient was waived by our institutional review board. Nevertheless, the patient records were anonymized and de-identified prior to analysis.

Although most patients (100 in 188 patients) were insulted with multiple injuries, TBI was the most severe trauma and accounted for the main reason for admission. TBI severity was determined on the basis of the lowest recorded Glasgow Coma Score (GCS) (mild, 13–15; moderate, 9–12; severe, 6–8; extreme severe, 3–5), length of coma (LOC) (mild, <30 min; moderate, 20 min to 6 h; severe, >6 h; extreme severe, persistence), and/or post-traumatic amnesia (PTA) (mild, <60 min; moderate, >60 min to <24 h; severe, ≥24 h), injury feature (mild, concussion; moderate, no cerebral compression; severe, diffuse cerebral injury; extremely severe, severe primary injury, decerebrate rigidity or other organ injury, shock). When the GCS, LOC, and PTA scores for a participant did not all fall into a single category, the patient was assigned to the most severe category. All these variables had been reported to have prognostic value and were measured by the experienced neurological surgeons on admission.

Variables used to assess comparability were age, sex, Acute Physiology and Chronic Health Evaluation II (APACHE II) score, baseline admission laboratory values, vital signs in the first 24 hours, and episode of infection from the hospital database. We used all-cause 90-day mortality as our primary endpoint, length of stay (LOS) in hospital and in intensive care unit (ICU) as the secondary outcomes.

### Control patients

To these patients we matched 50 healthy controls by gender and age. Healthy controls were under routine physical examination in the same hospital. Exclusion criteria included a history of cerebral or cardiac event during the previous 12 months, known inflammatory diseases, history of acute febrile disease or infection during the previous 3 months, known malignancy, pregnancy, and invasive procedures during the previous 6 months.

### Sample collection

Whole blood samples were obtained from all patients within 24 hours after hospital admission and up to 3 days after injury (mean 13.3+19.2 h after TBI onset), and were collected frequently during hospital stay. Samples were centrifuged at 2000 × g for 10 min at 4°C to pellet cellular bodies and debris. Patients were not included in the study if blood was not taken within 24 hours after admission or 72 hours after injury, or if patients were transferred from other hospitals.

Blood cell counts were performed with the Beckman Coulter 780 (Beckman Coulter, Nyon, Swiss). Total serum ChE activities including AChE and BChE were recorded for each patient. ChE activity was measured using reagent for creatine kinase test (Gcell, Jiuqiang Biotechnology, Beijing, China) with the Hitachi 7060 analyzer (Hitachi High-Technologies Corporation, Tokyo, Japan). The functional assay sensitivity (that is, the lowest concentration that can be quantified with a between-run imprecision of 0.030) met the Roche Diagnostics specification of 0.06 ng/mL. The respective within- and between-day coefficients of variation for ChE analyses were all less than 10%.

### Sepsis, neurocognition and neurofunction evaluation

Systemic inflammatory response syndrome (SIRS), sepsis, and severe sepsis were evaluated according to established criteria [34]. Sepsis was defined as a systemic response to an infection including the criteria for SIRS plus microbiological evidence of a focal infection and/or a positive blood culture. Septic shock was defined as sepsis-induced hypotension, persisting despite adequate fluid resuscitation, along with the presence of hypoperfusion abnormalities or organ dysfunction [35]. Patients were considered to suffer from SIRS if they met the criteria defined in the guidelines of the American College of Chest Physicians/Society of Critical Care Medicine [36].

The database included the cognitive function was assessed by the use of GCS [37,38] and the Mini-Mental State Examination (MMSE; score range, 0 to 30, with lower scores indicating poorer performance) [39]. In addition, observers blind to biomarker level evaluated the neurofunctional scores for the first 24 hours and global outcome at discharge using medical records retrospectively. The instruments for neurofunctional evaluation include the Glasgow Outcome Score (GOS) [40], Functional Independence Measure (FIM) [39], Disability Rating Scale (DRS-F) [41], Modified Rankin Scale (MRS) [42], Referral Decision Scale (RDS) [43] and Quality of Life Index (QLI) [44]. GOS is the oldest and most widely outcome measure [40] defined as follows: 1 = death; 2 = persistent vegetative state; 3 = severe disability; 4 = moderate disability; and 5 = good recovery [40]. The FIM has two scales, one including 13 motor items and another with 5 cognitive items. The scores describe the patient’s levels of independence in self-care, continence, mobility, communication, and cognition [45,46]. DRS is designed to reflect disability and handicap *via* evaluation of physical impairment and cognitive ability [47]. Higher score on the DRS indicates greater functional impairment, whereas the inverse is true for the FIM [43]. The functional outcome was determined by MRS [48,49]. The 14-item RDS is a psychiatric evaluation for mental disorder [43]. QLI is a short self-administered scale evaluating five aspects of quality of life: activity, daily living, health, support and outlook, with a choice of three possible answers and with higher scores reflecting a better quality of life [50].

### Statistical analysis

Continuous variables (ChE, age) were expressed as mean±SD. Continuous non-parametric variables (ICU and hospital stay, GCS, MMSE) were expressed as median (interquartile range, IQR). Categorical data were expressed as frequency and percentage. Continuous variables were compared between groups by the two-tailed Student *t*-test. We used the Chi-square test to compare categorical data and proportions, and Mann-Whitney U test as appropriate, to compare non-parametric data.

Spearman correlation coefficients were calculated to analyze the associations between admission ChE activity and the blood cell counts, cognitive and neurofunctional scores, as well as the hospital and ICU LOS. The Kruskal-Wallis test (non-parametric ANOVA) with post-hoc comparisons was utilized to identify differences in ChE levels, blood cell counts, APACHE II score, GCS and MMSE scores among the groups categorized by disease severity, infective status, or outcome. Dunn’s post-hoc tests were controlled for multiple comparisons. Sensitivity, specificity, and the receiver operating characteristic (ROC) curves were constructed and the areas under the curve (AUC) were also calculated.

The Binary logistic and Cox regression models (backward conditional step-wise) were used to identify the predicting factors (WBC, monocyte counts, neutrophil counts, MMSE, GCS, ChE, sepsis, hospital LOS, ICU LOS, and APACHE II) for death, adjusted for age and gender according to the established etiological basis for TBI. Then the variables that were significant different between the survivals and the non-survivals in the bivariable analysis were included in the final multivariable logistic regression analysis. All data in the present study were analyzed using SPSS version 16.0 (SPSS Inc, Chicago, USA).

## Results

### Enrollment

In this retrospective study, 15 initial ChE activities, 9 MMSE values, 5 lymphocyte counts, and 7 neutrophil counts were missing. Because the number of missing data was not so significant, they were ignored in the following statistic analysis. Patient demographics were shown in Table 1. Of the 188 TBI patients, 145 males and 33 females, with a mean age of 42.5 years (range 14–92), 42 (22.3%) died within 90 days after entry into the study. Patients were categorized by TBI severity as determined in the method with 23 mild brain trauma, 39 moderate brain trauma, 61 severe brain trauma, and 22 extremely severe brain trauma. At discharge, 32 patients had a GOS of 1 (death), 17 patient remained to be with a GOS of 2 (persistent vegetative state), 16 patients had a GOS of 3 (severe disability), 55 patients had a GOS of 4 (moderate disability), and 68 patients had a GOS of 5 (good recovery). No patients died within 3 days, whereas 10 (5.3%) died within 7 days, 18 (9.6%) died between 7–14 days, and 15 (8.0%) died between 14–31 days. Seventy-two (38.3%) patients developed infection, mostly in lungs (40, 55.6%), 38 (52.8%) of the infected patients died.

**Table 1:** Clinical characteristics of entire TBI patient cohort.

Variables		Total	Mild	Moderate	Severe	Extreme Severe	p value
		(n = 188)	(n = 34)	(n = 53)	(n = 72)	(n = 29)	
**age, year** (median±SEM)		42.50±1.51	37.50±3.77	46.00±3.20	41.5±2.05	48.00±3.96	0.228
**gender, male (n, %)**		145 (77.13)	23 (67.65)	39 (73.58)	61 (84.72)	22 (75.86)	0.213
**Diagnosis of TBI (n, %)**							
Skull fracture		107 (56.91)	17 (50.00)	32 (60.38)	33 (45.83)	18 (62.07)	0.293
Contusion		114 (60.64)	0 (0.00)	33 (62.26)	42 (58.33)	20 (68.97)	0.000
Primary injury	Subarachnoid hemorrhage	83 (44.15)	6 (17.65)	20 (37.74)	26 (36.11)	16 (55.17)	0.022
	Diffuse axonal injury	24 (12.77)	0 (0.00)	0 (0.00)	10 (13.89)	7 (24.14)	0.000
Second injury	Cerebral hernia	28 (14.89)	0 (0.00)	0 (0.00)	7 (9.72)	19 (65.52)	0.000
	Epidural hematoma	39 (20.74)	0 (0.00)	14 (26.42)	9 (12.50)	7 (24.14)	0.005
	Subdural hematoma	56 (29.79)	2 (5.88)	7 (13.21)	21 (29.17)	18 (62.07)	0.000
	Intracerebral hematoma	40 (21.28)	0 (0.00)	8 (15.09)	16 (22.22)	12 (41.38)	0.000
**MMSE** (median±SEM)		12.00±0.89	25.00±0.48	22.50 ±1.23	0±1.10	0.00±0.97	0.000
**APACHE‖**(median±SEM)		13.00±0.58	8.00±0.56	9.00±0.56	14.50±0.77	25.00±0.96	0.000
**GCS** (median±SEM)		13.00±0.332	15.00±0.04	15.00±0.17	8.00±0.43	4.00±0.25	0.000
**ICU LOS** (d,median±SEM)		5.00±5.83	2.20±1.32	3.00±9.91	7.00±13.12	8.00±4.71	0.000
**Hospital LOS** (d,median±SEM)		13.00±6.85	8.00±1.34	12.00±2.79	18.00±7.06	9.00±41.14	0.000
**Medication** (n, %)	Sedative	81 (43.09)	4 (11.76)	21 (39.62)	28 (38.89)	11 (37.93)	0.027
	Resuscitant analeptic	101 (53.72)	11 (32.35)	28 (52.83)	27 (37.50)	16 (55.17)	0.100
	Diuretic	139 (73.94)	6 (17.65)	37 (69.81)	45 (62.50)	28 (96.55)	0.000
	Hormone	111 (59.04)	2 (5.88)	21 (39.62)	42 (58.33)	27 (93.10)	0.000
	Ca^2+^ antagonist	60 (31.91)	6 (17.65)	18 (33.96)	14 (19.44)	12 (41.38)	0.046
	Vasodilator	8 (4.26)	0 (0.00)	6 (11.32)	5 (6.94)	7 (24.14)	0.009
	β-receptor blockers	29 (15.43)	2 (5.88)	6 (11.32)	5 (6.94)	4 (13.79)	0.587
**Mortality** (n, %)		34 (18.09)	0 (0.00)	1 (1.89)	18 (25)	22 (75.86)	0.000

All nonparametric data were presented as median values with standard error of mean (SEM). All categorical data were presented as absolute numbers and percentages.

On admission, the patients presented with either skull fracture or contusion, or both of them. The contusion was mostly seen in extremely severe patients, whereas the skull fracture occurred randomly among the groups. TBI was also categorized into primary injury (subarachnoid hemorrhage, diffuse axonal injury) and secondary injury (cerebral hernia, epidural hematoma, subdural hematoma and intracerebral hematoma). All the injuries were mostly noted in the extremely severe patients. As expected, APACHE II and cognitive scores (GCS and MMSE) were significantly different among various groups, so were the secondary outcomes (ICU LOS and hospital LOS). It should be noticed that the extremely severe patients had the longest ICU LOS, and the severe patients had the longest hospital LOS. It could be explained by the fact that the extremely severe patients had the highest mortality rate (75.86%), thus they stayed less time in hospital than the severe patients.

### ChE activity was acutely decreased in non-surviving, septic patients with severe injury

**Fig 1:**
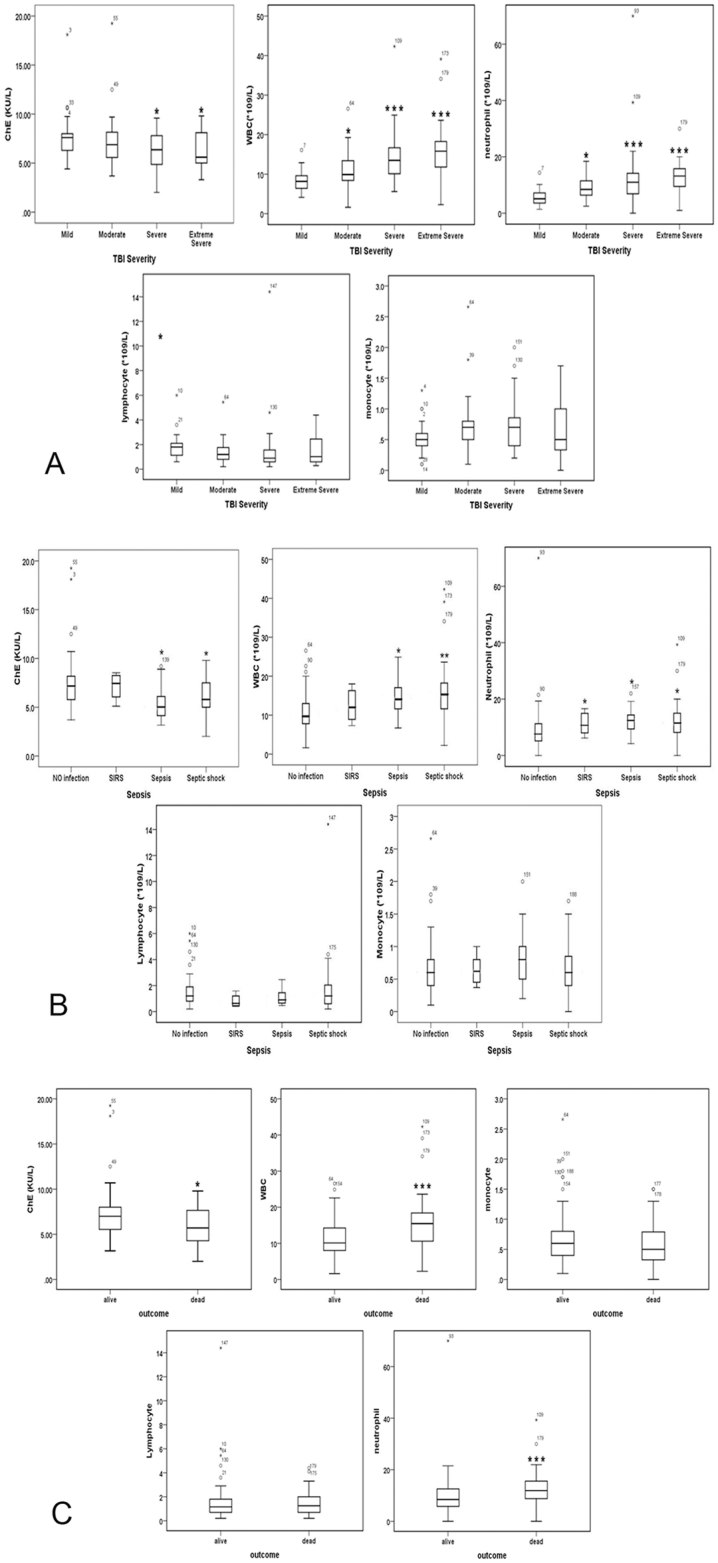
Admission ChE activity and blood cell counts in TBI patients as classified according to the severity (A), infected status (B), and the outcome (C). Boxes represented lower, median (line) and upper quartiles; whiskers showed the range of the data excluding outliers (。). *P<0.05, **P<0.01, ***P<0.001, compared with corresponding mild (A), non-infection (B) and survived (C) patients as analyzed by nonparametric test of several independent samples test (The Kruskal-Wallis H test).

Compared to matched controls, TBI patients showed lower serum AChE activity on admission (6.86±1.70 *vs*. 7.60±2.21, P = 0.029). To assess whether ChE activity was associated with the morbidity and mortality, subjects categorized by TBI severity, infected status (SIRS, sepsis, severe sepsis) and outcome (alive and dead) were analyzed by Post-hoc, Dunn’s multiple comparison tests following a Kruskal-Wallis test. Serum ChE activity on admission was significantly decreased in severe and extremely severe TBI patients as compared to that in mild patients. By contrast, both the white blood cell (WBC) counts and the neutrophil counts were elevated in groups of moderate, severe, and extremely severe patients in comparison to mild injured patients (Fig 1A). Similar results were seen in Fig 1B, in which ChE activity was significantly repressed in serum of infected patients as compared with non-infected patients. Non-survived patients also showed decreased ChE activity but increased WBC and neutrophil counts relative to survivors (Fig 1C). However, neither the lymphocyte nor the monocyte counts differed among groups of different categories.

### ChE activity was consistently depressed in non-survived, septic patients with severe injury

**Fig 2:**
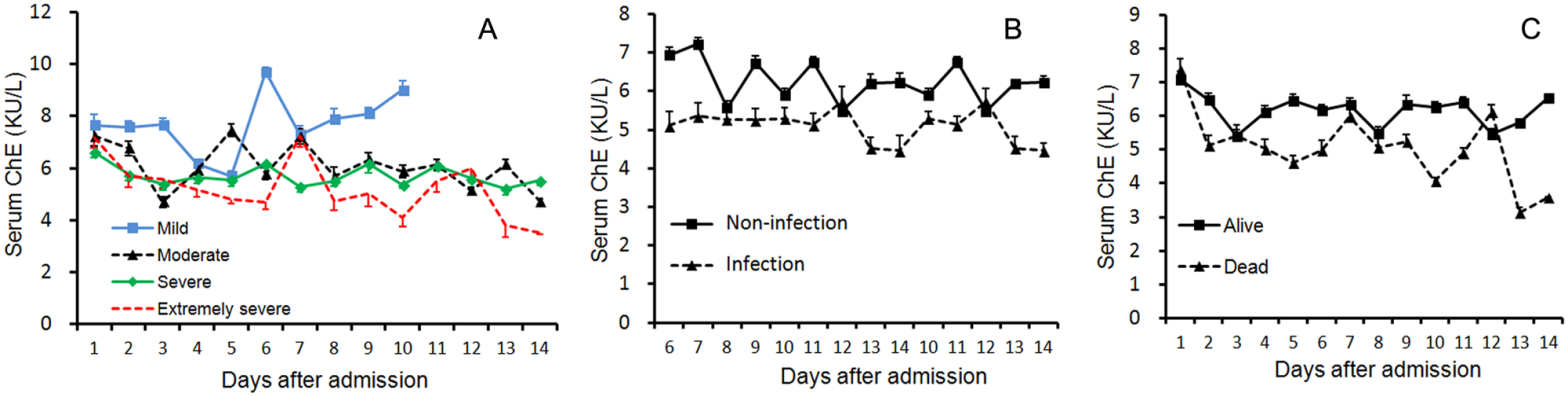
Kinetics of serum ChE activities in TBI patients categorized by disease severity (A), infected status (B), and outcome (C).

The trajectories of serum ChE activity in the first 2 weeks of injury were classified by TBI severity (Fig 2A), infected status (Fig 2B), and outcome (Fig 2C). ChE activity was decreased in mild TBI patients on day 4 post injury, then was elevated on day 6 until fully recovered on day 8. By contrast, in other three groups, serum ChE levels dropped sharply on day 2 post injury, and continued to decline during the observational period (Fig 2A). Likewise, septic or non-survived exhibited consistently lower levels of ChE relative to the corresponding non-infected or survived throughout their stay in hospital (Fig 2B and 2C).

### Association of ChE activity with clinical severity, neurocognitive and functional outcomes

Since serum ChE activities measured on admission were differentially reduced according to the severity, infected status and outcome, we wondered whether it could possibly be the diagnostic and prognostic biomarker for TBI. To this end, we examined the correlations of ChE activities with the blood cell counts, APACHE II score, cognitive and neurofunctional scores, and outcomes (ICU LOS, hospital LOS) in total population or in patients dichotomized by survival.

**Fig 3:**
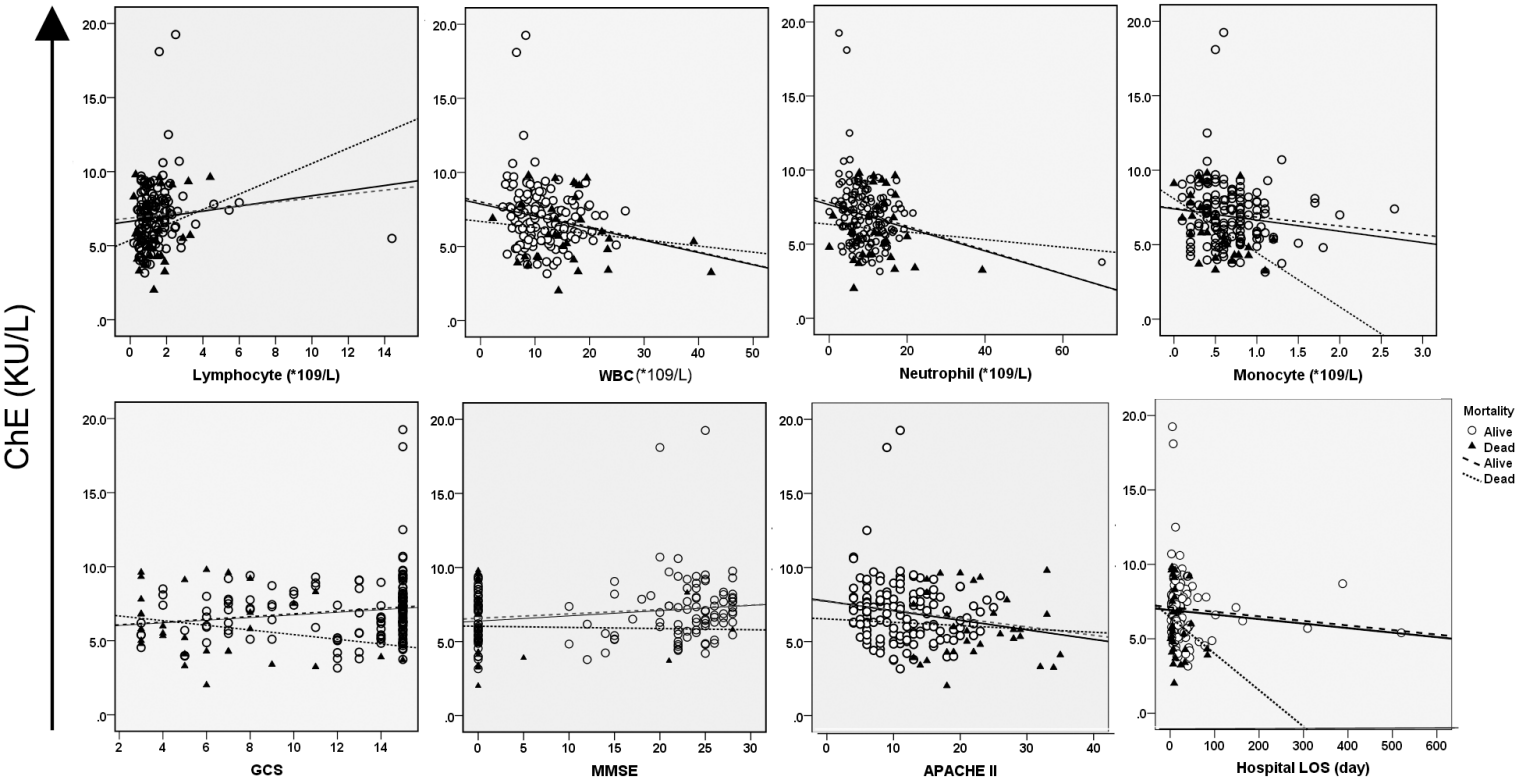
Scatterplot of the correlation of admission ChE activity with blood cell counts, neurocognitive scores, APACHE II score and hospital length of stay (LOS) in total population or in patients dichotomized by outcome.

The modified scatter plots of ChE activities against the above parameters were constructed in total patients or patients classified by survival status (Fig 3). A calculated linear regression line was drawn for each plot followed by the Spearman correlation analysis. Admission ChE activities were significantly correlated with blood cell counts, APACHE II score, cognition scores and ICU LOS in total population. However, most of the correlations in the whole population did not apply to the subgroups of patients dichotomized by survival status (Table 2). It was noteworthy that the admission ChE activities were correlated positively with lymphocyte counts, negatively with WBC, neutrophil and monocyte counts. It could be possible that as a source of ChE, lymphocytes may respond toward brain injury differently from other cell types. Taken together, ChE on admission was indicated as a stratified biomarker in disease onset, severity stage, and final outcomes.

**Table 2:** The correlation of serum ChE activity on admission with blood cell counts, neurocognitive scores, APACHE II score and hospital length of stay (LOS) in total or in patients with different outcome (Spearman’s correlation).

Outcome	Correlation	WBC	lymphocyte	neutrophil	monocyte	APACHE II	GCS	MMSE	hospital LOS	ICU LOS
All	Coefficiency	-0.156	0.208	-0.203	-0.171	-0.215	0.193	0.173	-0.147	-0.187
	P value	0.041	0.007	0.009	0.028	0.005	0.011	0.026	0.054	0.014
alive	Coefficiency	-0.155	0.241	-0.251	-0.137	-0.160	0.184	0.150	-0.165	-0.173
	P value	0.067	0.004	0.003	0.107	0.059	0.029	0.084	0.050	0.040
dead	Coefficiency	-0.018	0.090	0.066	-0.446	0.029	-0.319	-0.160	-0.227	-0.088
	P value	0.920	0.629	0.726	0.015	0.875	0.075	0.380	0.211	0.631

There were significant correlations between serum ChE activity and the above parameters in all population, but not always so significant in subgroups dichotomized by outcome.

The above findings prompted us to evaluate the potential value of admission ChE level in foreseeing the cognitive and neurofunctional outcomes of TBI patients. Interestingly, serum ChE activity on admission was significantly associated with the cognitive (GCS and MMSE) and neurofunctional scores (GOS, FIM, RDS, DRS-F, MRS, QLI) assessed both on admission (in) and at discharge (out) as analyzed by two-tailed Spearman rank test (Table 3). Similarly, the admission ChE activities were correlated more significantly (less P values) with the parameters obtained at discharge than on admission, suggesting its long-term prognostic value on TBI functional recovery.

**Table 3:** Association of admission ChE activity with neurocognitive (GCS, MMSE) and functional scores examined both on admission (in) and at discharge (out) in TBI patients (Spearman’s correlation).

Scales	GCS		MMSE		QLI		FIM		GOS		MRS		RDS		DRS-F	
**Time**	in	out	in	out	in	out	in	out	in	out	in	out	in	out	in	out
**Median**	13	15	12	25	4	7	70	118	3	4	4	2	10	3	4	3
**SEM**	0.342	0.365	0.894	0.888	0.195	0.271	3.479	3.776	0.064	0.113	0.112	0.142	0.771	0.848	0.093	0.848
**Coefficiency**	0.179	0.216	0.162	0.212	0.186	0.254	0.176	0.218	0.217	0.219	-0.15	-0.23	-0.17	-0.24	-0.17	-0.23
**P value**	0.017	0.004	0.036	0.006	0.013	0.001	0.019	0.004	0.004	0.003	0.043	0.002	0.022	0.002	0.022	0.003

It was revealed the positive correlations of ChE activity with neurocognitive scores (GCS, MMSE) and neurofunctional score (QLI, FIM, GOS), but negatively with impairment scores (MRS, RDS, DRS-F).

### ChE activity was not identified as the independent biomarker for mortality

To compare the importance of admission ChE activity along with other established markers in prediction of survival, both the bivariate analysis and multivariable analyses were performed. With bivariate logistic regression, using biomarkers as continuous independent variables, we found that ChE activity on admission and other variables such as the blood cell counts, cognitive scores, disease severity and infective status were significantly associated with 90-day mortality (Table 4). To avoid the influence caused by univariate analysis, a multivariable stepwise linear regression was also performed, taking into account the variables with clinical relevance (APACHE II), severity of illness (first hospital LOS, ICU LOS), neurocognitive scores (first GCS and MMSE), blood cell counts and the infective complication (sepsis) that were statistically significant in the bivariate analysis. As analyzed by Binary logistic regression after adjusting with age and gender, ChE on admission was not identified as an independent predictor for the probability of 90-day mortality (P = 0.078). However, the borderline p-value suggested that ChE on admission could possibly be used in predicting TBI mortality. Considering that some ChE values were missing, an different conclusion might be drawn if more data had been collected or multiple sampling had been met. Nevertheless, APACHE II score, sepsis, and ICU LOS were still independently associated with mortality (Table 4).

**Table 4:** Univariable and multivariable logistic regression model of prognosis the outcome at 90-days survival of TBI patients.

Variables	Univariable Analysis			Multivariable Analysis	
	Nonsurvival(n = 42)	Survived(n = 146)	P value	OR (95%CI)	P value
Age,year (Median,IQR)	47.00 (32.00~71.25)	41.00 (24.00~54.00)	0.011	1.015 (0.961~1.072)	0.589
APACHE II (Median,IQR)	22.50 (18.00~29.00)	11.0 (6.00~15.00)	0.000	1.316 (1.025~1.689)	0.031
GCS (Median,IQR)	5.00 (3.00~7.25)	15.0 (10.00~15.00)	0.000	0.846 (0.537~1.332)	0.469
MMSE (Median,IQR)	0.00 (0.00~0.00)	20.0 (0.00~25.00)	0.000	0.957 (0.848~1.079)	0.471
WBC,10^9^/L (Median,IQR)	15.47 (10.54~18.67)	10.10 (8.00~14.28)	0.000	1.081 (0.916~1.276)	0.359
Neutrophil,10^9^/L (Median,IQR)	11.92 (8.70~15.70)	8.40 (5.75~12.61)	0.000	1.008 (0.892~1.139)	0.899
Lymphocyte,10^9^/L (Median,IQR)	1.25 (0.65~2.05)	1.15 (0.70~1.80)	0.562	5.064 (1.216~21.038)	—
Monocyte,10^9^/L (Median,IQR)	0.50 (0.32~0.80)	0.60 (0.40~0.80)	0.137	0.003 (0.000~0.190)	—
ChE,KU/L (Median,IQR)	5.70 (4.27~7.80)	7.0 (5.50~8.00)	0.023	0.660 (0.416~1.047)	0.078
Hospital LOS,day (Median,IQR)	9.00 (5.00 ~17.50)	14.00 (9.00~25.25)	0.005	0.974 (0.936~1.011)	0.159
ICU LOS,day (Median,IQR)	6.5(4.00~ 11.75)	4.00 (2.00 ~10.00)	0.006	1.007 (1.000~1.013)	0.041
Sepsis, n (%)	38 (90.48%)	34 (23.29%)	0.000	1.819 (0.302~3.918)	0.048

Data were expressed as number and percentage, or median and 25th and 75th percentiles. Differences between survivors and non-survivors were evaluated by the Mann-Whitney U test. CI: Confidence interval.

### The predictive value of admission ChE activity for mortality

**Fig 4:**
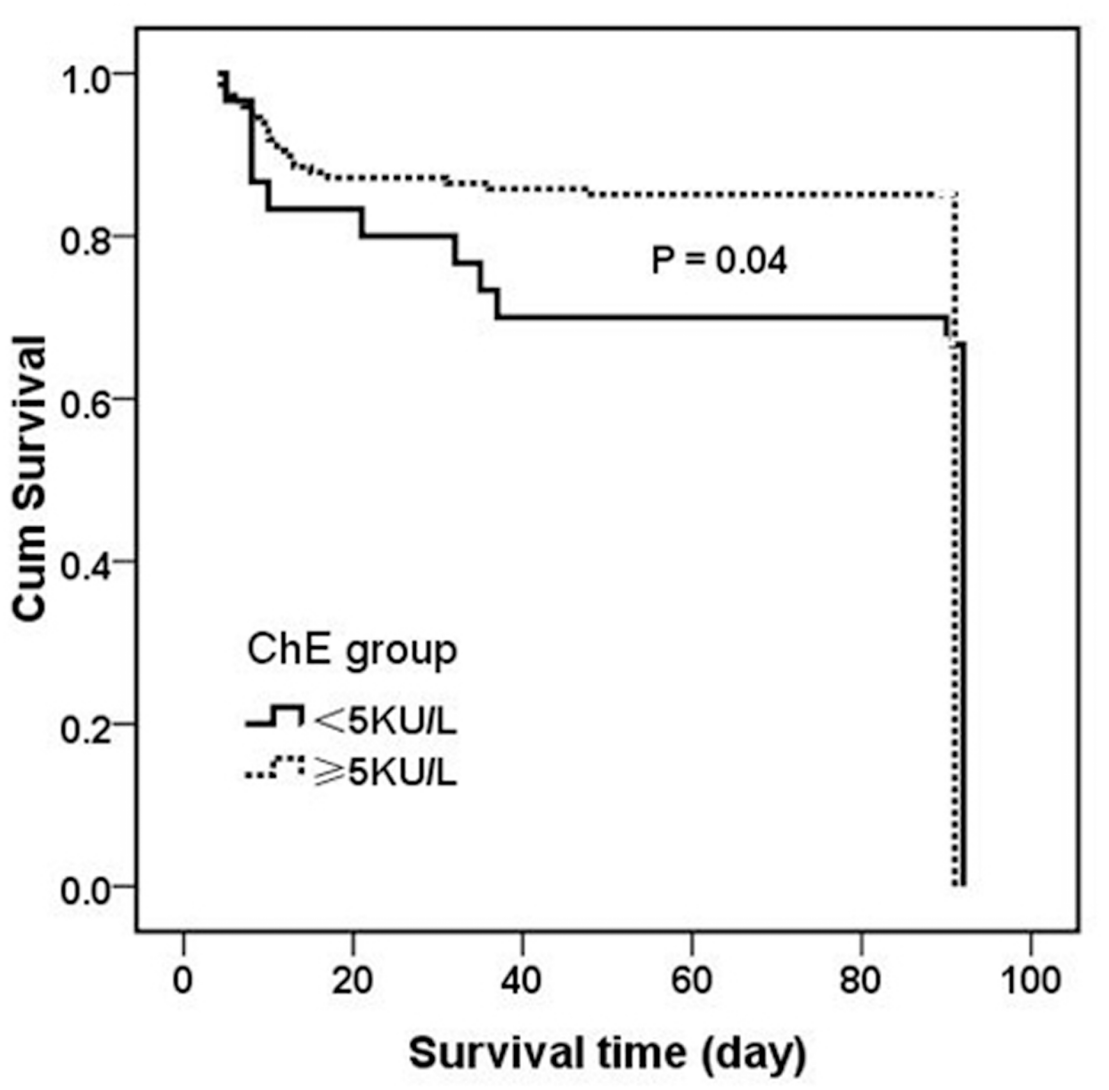
Kaplan-Meier analysis comparing the cumulative percentages of patients who survived according to their serum ChE activities measured 72 hours after TBI. Patients with serum ChE levels >5 kU/L showed a significantly higher chance to survive than patients with lower levels (log-rank test, p = 0.04).

Since the admission ChE activity was closely associated with the worst outcome, we wondered to what extent it was of any value in predicting the mortality. Receiver operating characteristic (ROC) analysis (S1 Fig) showed that area under the curve (AUC) for ChE was 0.381 (P = 0.046), which was inferior to that for either APACHE II (0.914, P = 0.000) or WBC (0.695, P = 0.001) (S1 Table). However, at the cutoff value of 5 kU/L, the sensitivity of ChE for correct prediction of death was 82.4% and the specificity was 92.2%. When patients were divided into two subcohorts according to the optimal threshold for serum ChE level in ROC analysis, a ChE level less than 5 kU/L (lower ChE group) was correlated markedly with poor overall patient survivals compared with a ChE level higher than 5 kU/L (higher ChE group) as indicated by Kaplan-Meier analysis (p = 0.04 by log-rank test) (Fig 4). The 90-day survival rates for patients with a ChE level<5 kU/L *vs*. ≥5 kU/L were 62% *vs*. 80%, respectively.

Additional Cox regression analysis revealed that only hospital LOS and sepsis were associated with the surviving days, with the Cox regression hazard risk ratio of 0.996 [p = 0.044, 95% confidence interval (CI): 0.993–1.000] and 1.311 (p = 0.005, 95% CI: 1.087–1.581), respectively. Once again, serum ChE activity on admission was not significantly associated with the surviving days with hazard risk ratio of 1.021 (p = 0.601, 95% CI: 0.945–1.102).

## Discussion

The cholinergic anti-inflammatory pathway has been recognized as a major regulatory component of stress response and was proposed to result in immune paralysis in TBI patients [33]. Our study identified prominent yet distinct correlations of on admission serum cholinergic parameter with TBI severity, infective status, survival, cognitive and neurofunctional outcomes, thus providing useful diagnostic and prognostic insights of TBI. Reduction in serum ChE activity and consequent increased ACh implied augmented cholinergic signaling after TBI, potentially serving as protection from uncontrolled inflammatory reactions.

Our study has several strengths in the following aspects. We for the first time found the acute decrease of serum ChE activity in the non-survivors *vs*. survivors after TBI. In addition to its neuroendocrinal effect as the pivotal neurotransmitter [24], accumulating evidence have demonstrated that ACh levels are also crucial for controlling immune response in both the brain and the peripheral tissues [51]. As AChE is a key contributor to sustain ACh level, several studies had shown dynamic changes in AChE activity in the brain after TBI [28,29]. Accordingly, the cerebellar expression of miR-132 which potentiated cholinergic anti-inflammatory signaling by targeting AChE [52] was down-regulated early after brain injury [28]. The physiological and pathological significance of cerebral AChE after brain damage remains to be identified. It is presumed that the initial stimulus induces excitation through ACh release, and feedback overexpression of AChE acts to dampen excessive neurotransmission back towards normal levels [6]. So far, only one study had identified serum ChE to be acutely decreased in 50 TBI patients, indicating severe damage and poor prognosis [32]. In accordance, the reduced serum ChE in our observation was also associated with TBI severity and mortality. Furthermore, consistent with previous study that serum cholinesterase activity could distinguish stroke patients from controls and predict 12-month mortality [14], admission ChE at cutoff level of 5 kU/L could also distinguish the survival from the non-survival in our TBI patients. In addition, the kinetics of serum ChE activity in survivals was also distinct from that in non-survivals. Although admission ChE did not reach its independent significance in predicting mortality, the borderline p-value still implied its prognostic value in clinic. Moreover, considering that the ChE data was from single blood test, multiple sampling might increase its specificity and sensitivity. As the reference, the trajectories of serum ChE activity could also predict the morbidity and mortality of TBI patients regardless of the treatment. Therefore, serum ChE activity should be followed for both diagnostic and prognostic purposes.

The decreased serum ChE level seemed to be in conflict with aforementioned increased AChE in the brain. We assume that serum AChE activity might reflect the cholinergic anti- inflammatory activity in response to TBI other than a neurotransmitter in the brain. Direct evidence of augmented sympathetic and attenuated parasympathetic drive was found in patients with brain damage in relation to the severity [53], thereby potentiating the pro-inflammatory response. Furthermore, serum AChE activity might potentially serve as predictor of risk of inflammatory response in healthy subjects [54] and patients with infective diseases [55,56]. Both *in vitro* and *in vivo*, AChE was substantially down-regulated after lipopolysaccharide challenge, suggesting that systemic reduction in AChE activity was an integral part of the post-inflammatory response [52]. Clinical study also showed that serum AChE activity was inversely and prominently correlated with inflammatory markers in post-stroke patients [14]. Our second intriguing finding was that serum ChE activity was decreased in patients with sepsis and septic shock in comparison to patients without septic complication. The trajectory of serum ChE activity also remained at lower level in septic *vs*. non-septic patients, further confirming serum ChE as a marker of infection [14]. In the current study, we determined the total ChE activities other than AChE alone in patients suffered from TBI. The summated ACh hydrolyzing activities of free AChE and BChE in the plasma might reflect the “Cholinergic Status” as a whole. Thus, a decrease serum ChE level in TBI patients might directly reflect the prominent inflammatory response toward TBI. Considering that sepsis was more frequently seen in non-survivors compared with survivors (90.5% *vs*. 23.3%), there would be possible a causal relationship among serum ChE level, sepsis and mortality.

The third highlight of the present study was that ChE activities were differentially reduced according to the disease severity. It was found that patients with severe and extremely severe TBI showed more reduction in ChE activity as opposed to patients with mild TBI, indicating that the extent of attenuation in cholinergic activity was related to TBI severity. Moreover, the trajectory of serum ChE activity could distinguish the patients of extremely severe TBI from the other three degrees of severity, implying that cholinergic activity was progressively differentiated according to injury severity.

TBI may lead to physical, emotional, intellectual and/or social changes for the survivors [57]. Our most encouraging finding was that ChE activity on admission was significantly associated with neurocognitive and functional scores assessed both on admission and at discharge, suggesting that serum ChE activity might act as a putative link to neurofunctional outcome following TBI. It is not uncommon that TBI patients may show cholinergic-dependent [58] transient delirium [59] and long-term cognitive decline [60]. As a support, serum AChE activity showed inverse, reciprocal associations with anxiety measures [54]. Thus, it was tempting to speculate the close relationship between serum ChE activity and the cognition scores (GCS, MMSE) assessed simultaneously on admission. However, it was extraordinarily surprising to notice that serum ChE level on admission was correlated to cognition scores more significantly at discharge than on admission. Since cholinergic depletion predisposes to development of acute cognitive deficits upon subsequent systemic inflammatory insult [61], the cholinergic hypothesis of delirium may be more relevant in patients with prior vulnerability in the cholinergic system [62]. Therefore, it was reasonable to speculate that admission ChE level that reflecting cholinergic signaling immediately after brain injury might be associated with the cognition outcome at discharge. Similar associations were also found between the serum ChE level on admission and the neurofunctional measures. The neurological outcome is mostly determined by the primary brain damage. Because admission serum ChE level may represent the parasympathetic activity following brain damage, the close association of admission serum ChE level with the neurological function scores was within expectation. For clinical practice, serum AChE activity could be a novel predictive parameter to improve and personalize TBI outcomes. Once again, serum ChE activity on admission was correlated highly with the neurofunctional scores at discharge than on admission, strongly supporting its prognostic potential for TBI recovery.

## Conclusions

Taken together, acute serum ChE decrease in response to TBI might facilitate chronic inflammatory response, resulting in secondary injury and poor outcome after TBI. The extent of reduction might potentially predict surviving possibility, guiding treatment and rehabilitation practice. Although it was not the independent predictor for mortality, admission ChE activity could be included in one of the biomarker panel for the prognosis of TBI mortality.

## Supporting Information

S1 Fig Receiver operating characteristic (ROC)of ChE and other variables. (TIF)

S1 File Statistic analysis in Table 1. (XLSX)

S2 File Healthy control. (XLSX)

S3 File Severity analysis in Fig 1A. (DOCX)

S4 File Sepsis analysis in Fig 1B. (DOCX)

S5 File Survival analysis in Fig 1C. (DOCX)

S6 File ChE kinetics. (XLSX)

S7 File Correlation analysis of ChE with other variables. (DOCX)

S8 File Correlation analysis of ChE with neurofunctional values. (XLSX)

S9 File Statistic analysis of ChE in COX Regression. (DOCX)

S10 File Multivariate analysis for ChE and other variables. (DOCX)

S1 Table AUC for ChE and other variables in ROC analysis. (DOCX)
