# Supplementary figures and images for: Serum Total Cholinesterase Activity on Admission Is Associated with Disease Severity and Outcome in Patients with Traumatic Brain Injury
Source: PLoS One. 2015 Jun 24;10(6):e0129082. doi: 10.1371/journal.pone.0129082 (PMC4479571; doi:10.1371/journal.pone.0129082)

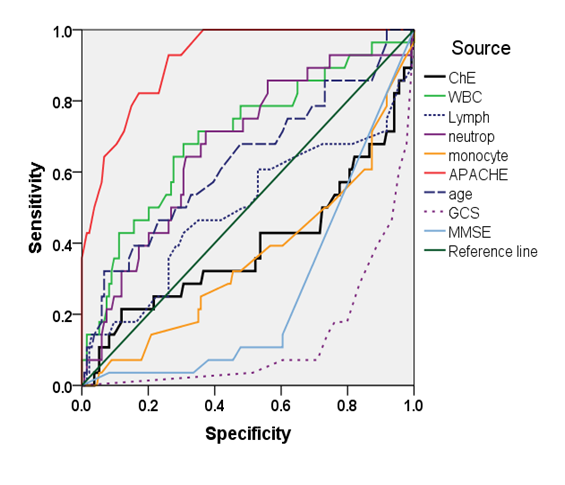

Supplement: S1 Fig — (TIF) [file pone.0129082.s001.tif]

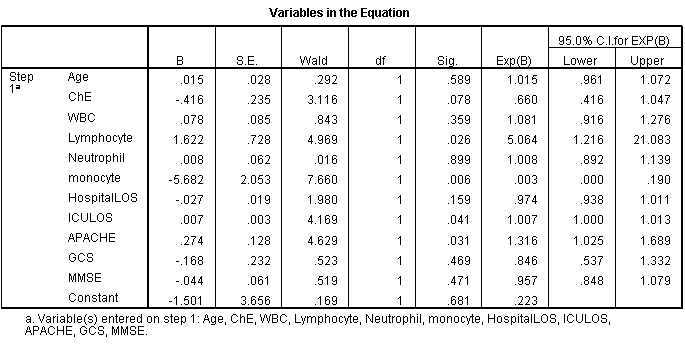

Supplement: S10 File — (DOCX) [file pone.0129082.s011.docx]
