# Supplementary material for: Serum Total Cholinesterase Activity on Admission Is Associated with Disease Severity and Outcome in Patients with Traumatic Brain Injury
Source: PLoS One. 2015 Jun 24;10(6):e0129082. doi: 10.1371/journal.pone.0129082 (PMC4479571; doi:10.1371/journal.pone.0129082)
Supplement: S3 File — (DOCX) [file pone.0129082.s004.docx]

**Kruskal-Wallis test**

| **Severity** | | | |
| --- | --- | --- | --- |
|  | Severity | N | Mean |
| ChE | 1 | 33 | 102.64 |
|  | 2 | 52 | 91.88 |
|  | 3 | 66 | 79.81 |
|  | 4 | 22 | 73.57 |
|  | Total | 173 |  |
| WBC | 1 | 34 | 46.96 |
|  | 2 | 52 | 81.24 |
|  | 3 | 71 | 109.39 |
|  | 4 | 28 | 129.18 |
|  | Total | 185 |  |
| Lymph | 1 | 33 | 120.42 |
|  | 2 | 52 | 93.13 |
|  | 3 | 70 | 77.95 |
|  | 4 | 28 | 91.52 |
|  | Total | 183 |  |
| neutrop | 1 | 33 | 40.67 |
|  | 2 | 53 | 84.51 |
|  | 3 | 66 | 106.86 |
|  | 4 | 29 | 124.05 |
|  | Total | 181 |  |
| lymphper | 1 | 33 | 139.92 |
|  | 2 | 53 | 99.34 |
|  | 3 | 70 | 72.29 |
|  | 4 | 28 | 74.18 |
|  | Total | 184 |  |
| monocyte | 1 | 33 | 71.30 |
|  | 2 | 53 | 100.97 |
|  | 3 | 68 | 98.46 |
|  | 4 | 29 | 84.00 |
|  | Total | 183 |  |
| hospitalLOS | 1 | 34 | 56.78 |
|  | 2 | 53 | 92.82 |
|  | 3 | 72 | 118.95 |
|  | 4 | 28 | 77.27 |
|  | Total | 187 |  |
| ICULOS | 1 | 34 | 75.37 |
|  | 2 | 53 | 64.19 |
|  | 3 | 72 | 112.99 |
|  | 4 | 29 | 126.43 |
|  | Total | 188 |  |
| APACHE | 1 | 34 | 47.85 |
|  | 2 | 53 | 61.89 |
|  | 3 | 72 | 112.16 |
|  | 4 | 28 | 164.13 |
|  | Total | 187 |  |
| age | 1 | 34 | 86.85 |
|  | 2 | 53 | 95.75 |
|  | 3 | 72 | 89.99 |
|  | 4 | 29 | 112.38 |
|  | Total | 188 |  |
| GCS | 1 | 34 | 147.85 |
|  | 2 | 53 | 132.80 |
|  | 3 | 72 | 70.31 |
|  | 4 | 29 | 22.00 |
|  | Total | 188 |  |
| MMSE | 1 | 34 | 144.68 |
|  | 2 | 53 | 120.11 |
|  | 3 | 72 | 68.83 |
|  | 4 | 29 | 52.60 |
|  | Total | 188 |  |

| **Non-parametric（K independent sample）test ^a,b^** | | | | | | | | | | | | |
| --- | --- | --- | --- | --- | --- | --- | --- | --- | --- | --- | --- | --- |
|  | ChE | WBC | Lymph | neutrop | lymphper | monocyte | hospitalLOS | ICULOS | APACHE | age | GCS | MMSE |
| Chi-square | 6.654 | 47.086 | 14.483 | 48.858 | 40.431 | 8.284 | 34.137 | 39.273 | 98.868 | 4.326 | 133.794 | 84.939 |
| df | 3 | 3 | 3 | 3 | 3 | 3 | 3 | 3 | 3 | 3 | 3 | 3 |
| Asymptotic significance | .084 | .000 | .002 | .000 | .000 | .040 | .000 | .000 | .000 | .228 | .000 | .000 |
| a. Kruskal Wallis test | | | | | | | | | | | | |
| b.variate groups：Severity | | | | | | | | | | | | |

| **One-way ANOVA** | | | | | | |
| --- | --- | --- | --- | --- | --- | --- |
|  | | Sum of square | df | Square of Mean | F | Sig |
| ChE | Between groups | 41.996 | 3 | 13.999 | 2.965 | .034 |
|  | In groups | 797.910 | 169 | 4.721 |  |  |
|  | Statistics | 839.906 | 172 |  |  |  |
| WBC | Between groups | 1293.577 | 3 | 431.192 | 15.615 | .000 |
|  | In groups | 4997.987 | 181 | 27.613 |  |  |
|  | Statistics | 6291.565 | 184 |  |  |  |
| Lymph | Between groups | 5.716 | 3 | 1.905 | 1.071 | .363 |
|  | In groups | 318.419 | 179 | 1.779 |  |  |
|  | Statistics | 324.134 | 182 |  |  |  |
| neutrop | Between groups | 1301.512 | 3 | 433.837 | 10.399 | .000 |
|  | In groups | 7384.634 | 177 | 41.721 |  |  |
|  | Statistics | 8686.146 | 180 |  |  |  |
| lymphper | Between groups | 4369.988 | 3 | 1456.663 | 14.332 | .000 |
|  | In groups | 18294.301 | 180 | 101.635 |  |  |
|  | Statistics | 22664.289 | 183 |  |  |  |
| monocyte | Between groups | .861 | 3 | .287 | 2.022 | .112 |
|  | In groups | 25.397 | 179 | .142 |  |  |
|  | Statistics | 26.258 | 182 |  |  |  |
| hospitalLOS | Between groups | 73604.311 | 3 | 24534.770 | 2.883 | .037 |
|  | In groups | 1557617.486 | 183 | 8511.571 |  |  |
|  | Statistics | 1631221.797 | 186 |  |  |  |
| ICULOS | Between groups | 26063.232 | 3 | 8687.744 | 1.368 | .254 |
|  | In groups | 1168806.944 | 184 | 6352.212 |  |  |
|  | Statistics | 1194870.176 | 187 |  |  |  |
| APACHE | Between groups | 5906.616 | 3 | 1968.872 | 73.813 | .000 |
|  | In groups | 4881.298 | 183 | 26.674 |  |  |
|  | Statistics | 10787.914 | 186 |  |  |  |
| age | Between groups | 2155.191 | 3 | 718.397 | 1.690 | .171 |
|  | In groups | 78220.836 | 184 | 425.113 |  |  |
|  | Statistics | 80376.026 | 187 |  |  |  |
| MMSE | Between groups | 12504.599 | 3 | 4168.200 | 55.231 | .000 |
|  | In groups | 13886.146 | 184 | 75.468 |  |  |
|  | Statistics | 26390.745 | 187 |  |  |  |
| GCS | Between groups | 2793.512 | 3 | 931.171 | 159.183 | .000 |
|  | In groups | 1076.339 | 184 | 5.850 |  |  |
|  | Statistics | 3869.851 | 187 |  |  |  |

**Post-hoc test**

| **Multiple comparation** | | | | | | | | |
| --- | --- | --- | --- | --- | --- | --- | --- | --- |
| Dependent variate | | (I) Severity | (J) Severity | Mean variation (I-J) | standard error | Significance | 95% CI | |
|  |  |  |  |  |  |  | low | high |
| ChE | LSD | 1 | 2 | .39171 | .48360 | .419 | -.5630 | 1.3464 |
|  |  |  | 3 | 1.15515^*^ | .46326 | .014 | .2406 | 2.0697 |
|  |  |  | 4 | 1.27545^*^ | .59806 | .034 | .0948 | 2.4561 |
|  |  | 2 | 1 | -.39171 | .48360 | .419 | -1.3464 | .5630 |
|  |  |  | 3 | .76344 | .40290 | .060 | -.0319 | 1.5588 |
|  |  |  | 4 | .88374 | .55263 | .112 | -.2072 | 1.9747 |
|  |  | 3 | 1 | -1.15515^*^ | .46326 | .014 | -2.0697 | -.2406 |
|  |  |  | 2 | -.76344 | .40290 | .060 | -1.5588 | .0319 |
|  |  |  | 4 | .12030 | .53492 | .822 | -.9357 | 1.1763 |
|  |  | 4 | 1 | -1.27545^*^ | .59806 | .034 | -2.4561 | -.0948 |
|  |  |  | 2 | -.88374 | .55263 | .112 | -1.9747 | .2072 |
|  |  |  | 3 | -.12030 | .53492 | .822 | -1.1763 | .9357 |
|  | Dunnett T3 | 1 | 2 | .39171 | .54526 | .977 | -1.0847 | 1.8681 |
|  |  |  | 3 | 1.15515 | .48956 | .123 | -.1825 | 2.4928 |
|  |  |  | 4 | 1.27545 | .60417 | .211 | -.3746 | 2.9255 |
|  |  | 2 | 1 | -.39171 | .54526 | .977 | -1.8681 | 1.0847 |
|  |  |  | 3 | .76344 | .40359 | .313 | -.3201 | 1.8469 |
|  |  |  | 4 | .88374 | .53687 | .479 | -.5867 | 2.3542 |
|  |  | 3 | 1 | -1.15515 | .48956 | .123 | -2.4928 | .1825 |
|  |  |  | 2 | -.76344 | .40359 | .313 | -1.8469 | .3201 |
|  |  |  | 4 | .12030 | .48020 | 1.000 | -1.2158 | 1.4564 |
|  |  | 4 | 1 | -1.27545 | .60417 | .211 | -2.9255 | .3746 |
|  |  |  | 2 | -.88374 | .53687 | .479 | -2.3542 | .5867 |
|  |  |  | 3 | -.12030 | .48020 | 1.000 | -1.4564 | 1.2158 |
| WBC | LSD | 1 | 2 | -2.731^*^ | 1.159 | .020 | -5.02 | -.44 |
|  |  |  | 3 | -5.673^*^ | 1.096 | .000 | -7.84 | -3.51 |
|  |  |  | 4 | -8.108^*^ | 1.341 | .000 | -10.75 | -5.46 |
|  |  | 2 | 1 | 2.731^*^ | 1.159 | .020 | .44 | 5.02 |
|  |  |  | 3 | -2.942^*^ | .959 | .002 | -4.83 | -1.05 |
|  |  |  | 4 | -5.377^*^ | 1.232 | .000 | -7.81 | -2.95 |
|  |  | 3 | 1 | 5.673^*^ | 1.096 | .000 | 3.51 | 7.84 |
|  |  |  | 2 | 2.942^*^ | .959 | .002 | 1.05 | 4.83 |
|  |  |  | 4 | -2.435^*^ | 1.173 | .039 | -4.75 | -.12 |
|  |  | 4 | 1 | 8.108^*^ | 1.341 | .000 | 5.46 | 10.75 |
|  |  |  | 2 | 5.377^*^ | 1.232 | .000 | 2.95 | 7.81 |
|  |  |  | 3 | 2.435^*^ | 1.173 | .039 | .12 | 4.75 |
|  | Dunnett T3 | 1 | 2 | -2.731^*^ | .734 | .002 | -4.71 | -.76 |
|  |  |  | 3 | -5.673^*^ | .819 | .000 | -7.87 | -3.48 |
|  |  |  | 4 | -8.108^*^ | 1.458 | .000 | -12.18 | -4.04 |
|  |  | 2 | 1 | 2.731^*^ | .734 | .002 | .76 | 4.71 |
|  |  |  | 3 | -2.942^*^ | .912 | .010 | -5.38 | -.51 |
|  |  |  | 4 | -5.377^*^ | 1.512 | .006 | -9.57 | -1.19 |
|  |  | 3 | 1 | 5.673^*^ | .819 | .000 | 3.48 | 7.87 |
|  |  |  | 2 | 2.942^*^ | .912 | .010 | .51 | 5.38 |
|  |  |  | 4 | -2.435 | 1.555 | .537 | -6.72 | 1.85 |
|  |  | 4 | 1 | 8.108^*^ | 1.458 | .000 | 4.04 | 12.18 |
|  |  |  | 2 | 5.377^*^ | 1.512 | .006 | 1.19 | 9.57 |
|  |  |  | 3 | 2.435 | 1.555 | .537 | -1.85 | 6.72 |
| Lymph | LSD | 1 | 2 | .438 | .297 | .142 | -.15 | 1.02 |
|  |  |  | 3 | .461 | .282 | .103 | -.09 | 1.02 |
|  |  |  | 4 | .223 | .343 | .517 | -.45 | .90 |
|  |  | 2 | 1 | -.438 | .297 | .142 | -1.02 | .15 |
|  |  |  | 3 | .023 | .244 | .924 | -.46 | .51 |
|  |  |  | 4 | -.215 | .313 | .492 | -.83 | .40 |
|  |  | 3 | 1 | -.461 | .282 | .103 | -1.02 | .09 |
|  |  |  | 2 | -.023 | .244 | .924 | -.51 | .46 |
|  |  |  | 4 | -.238 | .298 | .425 | -.83 | .35 |
|  |  | 4 | 1 | -.223 | .343 | .517 | -.90 | .45 |
|  |  |  | 2 | .215 | .313 | .492 | -.40 | .83 |
|  |  |  | 3 | .238 | .298 | .425 | -.35 | .83 |
|  | Dunnett T3 | 1 | 2 | .438 | .212 | .228 | -.14 | 1.01 |
|  |  |  | 3 | .461 | .273 | .444 | -.27 | 1.19 |
|  |  |  | 4 | .223 | .289 | .968 | -.57 | 1.01 |
|  |  | 2 | 1 | -.438 | .212 | .228 | -1.01 | .14 |
|  |  |  | 3 | .023 | .239 | 1.000 | -.62 | .66 |
|  |  |  | 4 | -.215 | .257 | .952 | -.92 | .49 |
|  |  | 3 | 1 | -.461 | .273 | .444 | -1.19 | .27 |
|  |  |  | 2 | -.023 | .239 | 1.000 | -.66 | .62 |
|  |  |  | 4 | -.238 | .309 | .968 | -1.07 | .60 |
|  |  | 4 | 1 | -.223 | .289 | .968 | -1.01 | .57 |
|  |  |  | 2 | .215 | .257 | .952 | -.49 | .92 |
|  |  |  | 3 | .238 | .309 | .968 | -.60 | 1.07 |
| neutrop | LSD | 1 | 2 | -3.595^*^ | 1.432 | .013 | -6.42 | -.77 |
|  |  |  | 3 | -6.745^*^ | 1.377 | .000 | -9.46 | -4.03 |
|  |  |  | 4 | -7.528^*^ | 1.644 | .000 | -10.77 | -4.28 |
|  |  | 2 | 1 | 3.595^*^ | 1.432 | .013 | .77 | 6.42 |
|  |  |  | 3 | -3.150^*^ | 1.191 | .009 | -5.50 | -.80 |
|  |  |  | 4 | -3.933^*^ | 1.492 | .009 | -6.88 | -.99 |
|  |  | 3 | 1 | 6.745^*^ | 1.377 | .000 | 4.03 | 9.46 |
|  |  |  | 2 | 3.150^*^ | 1.191 | .009 | .80 | 5.50 |
|  |  |  | 4 | -.783 | 1.439 | .587 | -3.62 | 2.06 |
|  |  | 4 | 1 | 7.528^*^ | 1.644 | .000 | 4.28 | 10.77 |
|  |  |  | 2 | 3.933^*^ | 1.492 | .009 | .99 | 6.88 |
|  |  |  | 3 | .783 | 1.439 | .587 | -2.06 | 3.62 |
|  | Dunnett T3 | 1 | 2 | -3.595^*^ | .699 | .000 | -5.48 | -1.71 |
|  |  |  | 3 | -6.745^*^ | 1.233 | .000 | -10.06 | -3.43 |
|  |  |  | 4 | -7.528^*^ | 1.101 | .000 | -10.57 | -4.49 |
|  |  | 2 | 1 | 3.595^*^ | .699 | .000 | 1.71 | 5.48 |
|  |  |  | 3 | -3.150 | 1.256 | .080 | -6.52 | .22 |
|  |  |  | 4 | -3.933^*^ | 1.126 | .007 | -7.03 | -.84 |
|  |  | 3 | 1 | 6.745^*^ | 1.233 | .000 | 3.43 | 10.06 |
|  |  |  | 2 | 3.150 | 1.256 | .080 | -.22 | 6.52 |
|  |  |  | 4 | -.783 | 1.517 | .996 | -4.86 | 3.30 |
|  |  | 4 | 1 | 7.528^*^ | 1.101 | .000 | 4.49 | 10.57 |
|  |  |  | 2 | 3.933^*^ | 1.126 | .007 | .84 | 7.03 |
|  |  |  | 3 | .783 | 1.517 | .996 | -3.30 | 4.86 |
| lymphper | LSD | 1 | 2 | 9.547^*^ | 2.236 | .000 | 5.14 | 13.96 |
|  |  |  | 3 | 13.690^*^ | 2.129 | .000 | 9.49 | 17.89 |
|  |  |  | 4 | 12.021^*^ | 2.590 | .000 | 6.91 | 17.13 |
|  |  | 2 | 1 | -9.547^*^ | 2.236 | .000 | -13.96 | -5.14 |
|  |  |  | 3 | 4.143^*^ | 1.836 | .025 | .52 | 7.77 |
|  |  |  | 4 | 2.474 | 2.355 | .295 | -2.17 | 7.12 |
|  |  | 3 | 1 | -13.690^*^ | 2.129 | .000 | -17.89 | -9.49 |
|  |  |  | 2 | -4.143^*^ | 1.836 | .025 | -7.77 | -.52 |
|  |  |  | 4 | -1.669 | 2.254 | .460 | -6.12 | 2.78 |
|  |  | 4 | 1 | -12.021^*^ | 2.590 | .000 | -17.13 | -6.91 |
|  |  |  | 2 | -2.474 | 2.355 | .295 | -7.12 | 2.17 |
|  |  |  | 3 | 1.669 | 2.254 | .460 | -2.78 | 6.12 |
|  | Dunnett T3 | 1 | 2 | 9.547^*^ | 2.603 | .003 | 2.47 | 16.62 |
|  |  |  | 3 | 13.690^*^ | 2.368 | .000 | 7.18 | 20.20 |
|  |  |  | 4 | 12.021^*^ | 3.095 | .002 | 3.61 | 20.43 |
|  |  | 2 | 1 | -9.547^*^ | 2.603 | .003 | -16.62 | -2.47 |
|  |  |  | 3 | 4.143 | 1.690 | .092 | -.40 | 8.68 |
|  |  |  | 4 | 2.474 | 2.613 | .917 | -4.67 | 9.62 |
|  |  | 3 | 1 | -13.690^*^ | 2.368 | .000 | -20.20 | -7.18 |
|  |  |  | 2 | -4.143 | 1.690 | .092 | -8.68 | .40 |
|  |  |  | 4 | -1.669 | 2.379 | .979 | -8.26 | 4.92 |
|  |  | 4 | 1 | -12.021^*^ | 3.095 | .002 | -20.43 | -3.61 |
|  |  |  | 2 | -2.474 | 2.613 | .917 | -9.62 | 4.67 |
|  |  |  | 3 | 1.669 | 2.379 | .979 | -4.92 | 8.26 |
| monocyte | LSD | 1 | 2 | -.190^*^ | .084 | .024 | -.36 | -.03 |
|  |  |  | 3 | -.173^*^ | .080 | .032 | -.33 | -.02 |
|  |  |  | 4 | -.125 | .096 | .193 | -.31 | .06 |
|  |  | 2 | 1 | .190^*^ | .084 | .024 | .03 | .36 |
|  |  |  | 3 | .018 | .069 | .799 | -.12 | .15 |
|  |  |  | 4 | .065 | .087 | .455 | -.11 | .24 |
|  |  | 3 | 1 | .173^*^ | .080 | .032 | .02 | .33 |
|  |  |  | 2 | -.018 | .069 | .799 | -.15 | .12 |
|  |  |  | 4 | .048 | .084 | .570 | -.12 | .21 |
|  |  | 4 | 1 | .125 | .096 | .193 | -.06 | .31 |
|  |  |  | 2 | -.065 | .087 | .455 | -.24 | .11 |
|  |  |  | 3 | -.048 | .084 | .570 | -.21 | .12 |
|  | Dunnett T3 | 1 | 2 | -.190 | .071 | .053 | -.38 | .00 |
|  |  |  | 3 | -.173^*^ | .063 | .041 | -.34 | .00 |
|  |  |  | 4 | -.125 | .096 | .724 | -.39 | .14 |
|  |  | 2 | 1 | .190 | .071 | .053 | .00 | .38 |
|  |  |  | 3 | .018 | .071 | 1.000 | -.17 | .21 |
|  |  |  | 4 | .065 | .102 | .987 | -.21 | .34 |
|  |  | 3 | 1 | .173^*^ | .063 | .041 | .00 | .34 |
|  |  |  | 2 | -.018 | .071 | 1.000 | -.21 | .17 |
|  |  |  | 4 | .048 | .096 | .997 | -.22 | .31 |
|  |  | 4 | 1 | .125 | .096 | .724 | -.14 | .39 |
|  |  |  | 2 | -.065 | .102 | .987 | -.34 | .21 |
|  |  |  | 3 | -.048 | .096 | .997 | -.31 | .22 |
| hospitalLOS | LSD | 1 | 2 | -7.996 | 20.272 | .694 | -47.99 | 32.00 |
|  |  |  | 3 | -27.814 | 19.198 | .149 | -65.69 | 10.06 |
|  |  |  | 4 | -61.790^*^ | 23.544 | .009 | -108.24 | -15.34 |
|  |  | 2 | 1 | 7.996 | 20.272 | .694 | -32.00 | 47.99 |
|  |  |  | 3 | -19.818 | 16.698 | .237 | -52.76 | 13.13 |
|  |  |  | 4 | -53.794^*^ | 21.554 | .013 | -96.32 | -11.27 |
|  |  | 3 | 1 | 27.814 | 19.198 | .149 | -10.06 | 65.69 |
|  |  |  | 2 | 19.818 | 16.698 | .237 | -13.13 | 52.76 |
|  |  |  | 4 | -33.976 | 20.548 | .100 | -74.52 | 6.56 |
|  |  | 4 | 1 | 61.790^*^ | 23.544 | .009 | 15.34 | 108.24 |
|  |  |  | 2 | 53.794^*^ | 21.554 | .013 | 11.27 | 96.32 |
|  |  |  | 3 | 33.976 | 20.548 | .100 | -6.56 | 74.52 |
|  | Dunnett T3 | 1 | 2 | -7.996 | 3.092 | .067 | -16.35 | .35 |
|  |  |  | 3 | -27.814^*^ | 7.185 | .001 | -47.20 | -8.43 |
|  |  |  | 4 | -61.790 | 41.160 | .586 | -178.05 | 54.47 |
|  |  | 2 | 1 | 7.996 | 3.092 | .067 | -.35 | 16.35 |
|  |  |  | 3 | -19.818 | 7.590 | .061 | -40.21 | .57 |
|  |  |  | 4 | -53.794 | 41.233 | .722 | -170.20 | 62.62 |
|  |  | 3 | 1 | 27.814^*^ | 7.185 | .001 | 8.43 | 47.20 |
|  |  |  | 2 | 19.818 | 7.590 | .061 | -.57 | 40.21 |
|  |  |  | 4 | -33.976 | 41.740 | .956 | -151.42 | 83.47 |
|  |  | 4 | 1 | 61.790 | 41.160 | .586 | -54.47 | 178.05 |
|  |  |  | 2 | 53.794 | 41.233 | .722 | -62.62 | 170.20 |
|  |  |  | 3 | 33.976 | 41.740 | .956 | -83.47 | 151.42 |
| ICULOS | LSD | 1 | 2 | -7.713 | 17.512 | .660 | -42.26 | 26.84 |
|  |  |  | 3 | -29.522 | 16.585 | .077 | -62.24 | 3.20 |
|  |  |  | 4 | -11.182 | 20.146 | .580 | -50.93 | 28.57 |
|  |  | 2 | 1 | 7.713 | 17.512 | .660 | -26.84 | 42.26 |
|  |  |  | 3 | -21.809 | 14.425 | .132 | -50.27 | 6.65 |
|  |  |  | 4 | -3.468 | 18.409 | .851 | -39.79 | 32.85 |
|  |  | 3 | 1 | 29.522 | 16.585 | .077 | -3.20 | 62.24 |
|  |  |  | 2 | 21.809 | 14.425 | .132 | -6.65 | 50.27 |
|  |  |  | 4 | 18.341 | 17.529 | .297 | -16.24 | 52.92 |
|  |  | 4 | 1 | 11.182 | 20.146 | .580 | -28.57 | 50.93 |
|  |  |  | 2 | 3.468 | 18.409 | .851 | -32.85 | 39.79 |
|  |  |  | 3 | -18.341 | 17.529 | .297 | -52.92 | 16.24 |
|  | Dunnett T3 | 1 | 2 | -7.713 | 9.997 | .968 | -34.97 | 19.54 |
|  |  |  | 3 | -29.522 | 13.173 | .155 | -65.10 | 6.06 |
|  |  |  | 4 | -11.182 | 4.893 | .156 | -24.84 | 2.48 |
|  |  | 2 | 1 | 7.713 | 9.997 | .968 | -19.54 | 34.97 |
|  |  |  | 3 | -21.809 | 16.431 | .706 | -65.72 | 22.11 |
|  |  |  | 4 | -3.468 | 10.973 | 1.000 | -33.12 | 26.18 |
|  |  | 3 | 1 | 29.522 | 13.173 | .155 | -6.06 | 65.10 |
|  |  |  | 2 | 21.809 | 16.431 | .706 | -22.11 | 65.72 |
|  |  |  | 4 | 18.341 | 13.928 | .713 | -19.12 | 55.80 |
|  |  | 4 | 1 | 11.182 | 4.893 | .156 | -2.48 | 24.84 |
|  |  |  | 2 | 3.468 | 10.973 | 1.000 | -26.18 | 33.12 |
|  |  |  | 3 | -18.341 | 13.928 | .713 | -55.80 | 19.12 |
| APACHE | LSD | 1 | 2 | -1.488 | 1.135 | .191 | -3.73 | .75 |
|  |  |  | 3 | -7.788^*^ | 1.075 | .000 | -9.91 | -5.67 |
|  |  |  | 4 | -16.977^*^ | 1.318 | .000 | -19.58 | -14.38 |
|  |  | 2 | 1 | 1.488 | 1.135 | .191 | -.75 | 3.73 |
|  |  |  | 3 | -6.300^*^ | .935 | .000 | -8.14 | -4.46 |
|  |  |  | 4 | -15.489^*^ | 1.207 | .000 | -17.87 | -13.11 |
|  |  | 3 | 1 | 7.788^*^ | 1.075 | .000 | 5.67 | 9.91 |
|  |  |  | 2 | 6.300^*^ | .935 | .000 | 4.46 | 8.14 |
|  |  |  | 4 | -9.188^*^ | 1.150 | .000 | -11.46 | -6.92 |
|  |  | 4 | 1 | 16.977^*^ | 1.318 | .000 | 14.38 | 19.58 |
|  |  |  | 2 | 15.489^*^ | 1.207 | .000 | 13.11 | 17.87 |
|  |  |  | 3 | 9.188^*^ | 1.150 | .000 | 6.92 | 11.46 |
|  | Dunnett T3 | 1 | 2 | -1.488 | .787 | .315 | -3.61 | .63 |
|  |  |  | 3 | -7.788^*^ | .946 | .000 | -10.32 | -5.25 |
|  |  |  | 4 | -16.977^*^ | 1.106 | .000 | -20.02 | -13.94 |
|  |  | 2 | 1 | 1.488 | .787 | .315 | -.63 | 3.61 |
|  |  |  | 3 | -6.300^*^ | .946 | .000 | -8.83 | -3.77 |
|  |  |  | 4 | -15.489^*^ | 1.106 | .000 | -18.52 | -12.45 |
|  |  | 3 | 1 | 7.788^*^ | .946 | .000 | 5.25 | 10.32 |
|  |  |  | 2 | 6.300^*^ | .946 | .000 | 3.77 | 8.83 |
|  |  |  | 4 | -9.188^*^ | 1.224 | .000 | -12.51 | -5.87 |
|  |  | 4 | 1 | 16.977^*^ | 1.106 | .000 | 13.94 | 20.02 |
|  |  |  | 2 | 15.489^*^ | 1.106 | .000 | 12.45 | 18.52 |
|  |  |  | 3 | 9.188^*^ | 1.224 | .000 | 5.87 | 12.51 |
| age | LSD | 1 | 2 | -2.546 | 4.530 | .575 | -11.48 | 6.39 |
|  |  |  | 3 | -1.078 | 4.290 | .802 | -9.54 | 7.39 |
|  |  |  | 4 | -10.395^*^ | 5.212 | .048 | -20.68 | -.11 |
|  |  | 2 | 1 | 2.546 | 4.530 | .575 | -6.39 | 11.48 |
|  |  |  | 3 | 1.467 | 3.732 | .695 | -5.90 | 8.83 |
|  |  |  | 4 | -7.849 | 4.762 | .101 | -17.24 | 1.55 |
|  |  | 3 | 1 | 1.078 | 4.290 | .802 | -7.39 | 9.54 |
|  |  |  | 2 | -1.467 | 3.732 | .695 | -8.83 | 5.90 |
|  |  |  | 4 | -9.316^*^ | 4.535 | .041 | -18.26 | -.37 |
|  |  | 4 | 1 | 10.395^*^ | 5.212 | .048 | .11 | 20.68 |
|  |  |  | 2 | 7.849 | 4.762 | .101 | -1.55 | 17.24 |
|  |  |  | 3 | 9.316^*^ | 4.535 | .041 | .37 | 18.26 |
|  | Dunnett T3 | 1 | 2 | -2.546 | 4.940 | .996 | -15.89 | 10.79 |
|  |  |  | 3 | -1.078 | 4.286 | 1.000 | -12.77 | 10.61 |
|  |  |  | 4 | -10.395 | 5.465 | .312 | -25.24 | 4.45 |
|  |  | 2 | 1 | 2.546 | 4.940 | .996 | -10.79 | 15.89 |
|  |  |  | 3 | 1.467 | 3.796 | .999 | -8.73 | 11.66 |
|  |  |  | 4 | -7.849 | 5.091 | .551 | -21.66 | 5.96 |
|  |  | 3 | 1 | 1.078 | 4.286 | 1.000 | -10.61 | 12.77 |
|  |  |  | 2 | -1.467 | 3.796 | .999 | -11.66 | 8.73 |
|  |  |  | 4 | -9.316 | 4.458 | .223 | -21.57 | 2.94 |
|  |  | 4 | 1 | 10.395 | 5.465 | .312 | -4.45 | 25.24 |
|  |  |  | 2 | 7.849 | 5.091 | .551 | -5.96 | 21.66 |
|  |  |  | 3 | 9.316 | 4.458 | .223 | -2.94 | 21.57 |
| MMSE | LSD | 1 | 2 | 5.058^*^ | 1.909 | .009 | 1.29 | 8.82 |
|  |  |  | 3 | 17.641^*^ | 1.808 | .000 | 14.07 | 21.21 |
|  |  |  | 4 | 21.829^*^ | 2.196 | .000 | 17.50 | 26.16 |
|  |  | 2 | 1 | -5.058^*^ | 1.909 | .009 | -8.82 | -1.29 |
|  |  |  | 3 | 12.583^*^ | 1.572 | .000 | 9.48 | 15.69 |
|  |  |  | 4 | 16.770^*^ | 2.007 | .000 | 12.81 | 20.73 |
|  |  | 3 | 1 | -17.641^*^ | 1.808 | .000 | -21.21 | -14.07 |
|  |  |  | 2 | -12.583^*^ | 1.572 | .000 | -15.69 | -9.48 |
|  |  |  | 4 | 4.187^*^ | 1.911 | .030 | .42 | 7.96 |
|  |  | 4 | 1 | -21.829^*^ | 2.196 | .000 | -26.16 | -17.50 |
|  |  |  | 2 | -16.770^*^ | 2.007 | .000 | -20.73 | -12.81 |
|  |  |  | 3 | -4.187^*^ | 1.911 | .030 | -7.96 | -.42 |
|  | Dunnett T3 | 1 | 2 | 5.058 | 1.896 | .053 | -.05 | 10.16 |
|  |  |  | 3 | 17.641^*^ | 1.702 | .000 | 13.05 | 22.23 |
|  |  |  | 4 | 21.829^*^ | 1.627 | .000 | 17.40 | 26.25 |
|  |  | 2 | 1 | -5.058 | 1.896 | .053 | -10.16 | .05 |
|  |  |  | 3 | 12.583^*^ | 1.748 | .000 | 7.90 | 17.27 |
|  |  |  | 4 | 16.770^*^ | 1.676 | .000 | 12.25 | 21.29 |
|  |  | 3 | 1 | -17.641^*^ | 1.702 | .000 | -22.23 | -13.05 |
|  |  |  | 2 | -12.583^*^ | 1.748 | .000 | -17.27 | -7.90 |
|  |  |  | 4 | 4.187^*^ | 1.453 | .029 | .28 | 8.09 |
|  |  | 4 | 1 | -21.829^*^ | 1.627 | .000 | -26.25 | -17.40 |
|  |  |  | 2 | -16.770^*^ | 1.676 | .000 | -21.29 | -12.25 |
|  |  |  | 3 | -4.187^*^ | 1.453 | .029 | -8.09 | -.28 |
| GCS | LSD | 1 | 2 | .620 | .531 | .245 | -.43 | 1.67 |
|  |  |  | 3 | 5.913^*^ | .503 | .000 | 4.92 | 6.91 |
|  |  |  | 4 | 10.907^*^ | .611 | .000 | 9.70 | 12.11 |
|  |  | 2 | 1 | -.620 | .531 | .245 | -1.67 | .43 |
|  |  |  | 3 | 5.293^*^ | .438 | .000 | 4.43 | 6.16 |
|  |  |  | 4 | 10.286^*^ | .559 | .000 | 9.18 | 11.39 |
|  |  | 3 | 1 | -5.913^*^ | .503 | .000 | -6.91 | -4.92 |
|  |  |  | 2 | -5.293^*^ | .438 | .000 | -6.16 | -4.43 |
|  |  |  | 4 | 4.993^*^ | .532 | .000 | 3.94 | 6.04 |
|  |  | 4 | 1 | -10.907^*^ | .611 | .000 | -12.11 | -9.70 |
|  |  |  | 2 | -10.286^*^ | .559 | .000 | -11.39 | -9.18 |
|  |  |  | 3 | -4.993^*^ | .532 | .000 | -6.04 | -3.94 |
|  | Dunnett T3 | 1 | 2 | .620^*^ | .171 | .003 | .16 | 1.08 |
|  |  |  | 3 | 5.913^*^ | .433 | .000 | 4.74 | 7.08 |
|  |  |  | 4 | 10.907^*^ | .249 | .000 | 10.21 | 11.61 |
|  |  | 2 | 1 | -.620^*^ | .171 | .003 | -1.08 | -.16 |
|  |  |  | 3 | 5.293^*^ | .462 | .000 | 4.05 | 6.53 |
|  |  |  | 4 | 10.286^*^ | .296 | .000 | 9.48 | 11.09 |
|  |  | 3 | 1 | -5.913^*^ | .433 | .000 | -7.08 | -4.74 |
|  |  |  | 2 | -5.293^*^ | .462 | .000 | -6.53 | -4.05 |
|  |  |  | 4 | 4.993^*^ | .496 | .000 | 3.66 | 6.32 |
|  |  | 4 | 1 | -10.907^*^ | .249 | .000 | -11.61 | -10.21 |
|  |  |  | 2 | -10.286^*^ | .296 | .000 | -11.09 | -9.48 |
|  |  |  | 3 | -4.993^*^ | .496 | .000 | -6.32 | -3.66 |
| *. 平均值差異在 0.05 層級顯著。 | | | | | | | | |

**同質子集**

| **ChE** | | | |
| --- | --- | --- | --- |
|  | Severity | N | alpha = 0.05 的子集 |
|  |  |  | 1 |
| Student-Newman-Keuls multiple comparation^a,b^ | 4 | 22 | 6.2982 |
|  | 3 | 66 | 6.4185 |
|  | 2 | 52 | 7.1819 |
|  | 1 | 33 | 7.5736 |
|  | significance |  | .063 |
|  | | | |
|  | | | |
|  | | | |

| **WBC** | | | | | | |
| --- | --- | --- | --- | --- | --- | --- |
|  | Severity | N | alpha = 0.05 subset | | | |
|  |  |  | 1 | 2 | 3 | 4 |
| Student-Newman-Keuls comparation^a,b^ | 1 | 34 | 8.25 |  |  |  |
|  | 2 | 52 |  | 10.98 |  |  |
|  | 3 | 71 |  |  | 13.93 |  |
|  | 4 | 28 |  |  |  | 16.36 |
|  | significance |  | 1.000 | 1.000 | 1.000 | 1.000 |

| **Lymph** | | | |
| --- | --- | --- | --- |
|  | Severity | N | alpha = 0.05 subset |
|  |  |  | 1 |
| Student-Newman-Keuls comparation^a,b^ | 3 | 70 | 1.32 |
|  | 2 | 52 | 1.34 |
|  | 4 | 28 | 1.56 |
|  | 1 | 33 | 1.78 |
|  | significance |  | .410 |

| **neutrophil** | | | | | |
| --- | --- | --- | --- | --- | --- |
|  | Severity | N | alpha = 0.05 subset | | |
|  |  |  | 1 | 2 | 3 |
| Student-Newman-Keuls comparation^a,b^ | 1 | 33 | 5.44 |  |  |
|  | 2 | 53 |  | 9.03 |  |
|  | 3 | 66 |  |  | 12.18 |
|  | 4 | 29 |  |  | 12.96 |
|  | significance |  | 1.000 | 1.000 | .586 |

| **Lymphocyte percentage** | | | | |
| --- | --- | --- | --- | --- |
|  | Severity | N | alpha = 0.05 subset | |
|  |  |  | 1 | 2 |
| Student-Newman-Keuls comparation^a,b^ | 3 | 70 | 9.73 |  |
|  | 4 | 28 | 11.40 |  |
|  | 2 | 53 | 13.88 |  |
|  | 1 | 33 |  | 23.42 |
|  | significance |  | .158 | 1.000 |
|  | | | | |
|  | | | | |
|  | | | | |

| **monocyte** | | | |
| --- | --- | --- | --- |
|  | Severity | N | alpha = 0.05 subset |
|  |  |  | 1 |
| Student-Newman-Keuls comparation^a,b^ | 1 | 33 | .53 |
|  | 4 | 29 | .66 |
|  | 3 | 68 | .70 |
|  | 2 | 53 | .72 |
|  | significance |  | .107 |

| **Hospital LOS** | | | | |
| --- | --- | --- | --- | --- |
|  | Severity | N | alpha = 0.05 subset | |
|  |  |  | 1 | 2 |
| Student-Newman-Keuls comparation^a,b^ | 1 | 34 | 9.85 |  |
|  | 2 | 53 | 17.85 |  |
|  | 3 | 72 | 37.67 | 37.67 |
|  | 4 | 28 |  | 71.64 |
|  | significance |  | .363 | .098 |

| **ICU LOS** | | | |
| --- | --- | --- | --- |
|  | Severity | N | alpha = 0.05 subset |
|  |  |  | 1 |
| Student-Newman-Keuls comparation^a,b^ | 1 | 34 | 5.85 |
|  | 2 | 53 | 13.57 |
|  | 4 | 29 | 17.03 |
|  | 3 | 72 | 35.38 |
|  | significance |  | .335 |

| **APACHE II** | | | | | |
| --- | --- | --- | --- | --- | --- |
|  | Severity | N | alpha = 0.05 subset | | |
|  |  |  | 1 | 2 | 3 |
| Student-Newman-Keuls comparation^a,b^ | 1 | 34 | 8.06 |  |  |
|  | 2 | 53 | 9.55 |  |  |
|  | 3 | 72 |  | 15.85 |  |
|  | 4 | 28 |  |  | 25.04 |
|  | significance |  | .194 | 1.000 | 1.000 |

| **age** | | | |
| --- | --- | --- | --- |
|  | Severity | N | alpha = 0.05 subset |
|  |  |  | 1 |
| Student-Newman-Keuls comparation^a,b^ | 1 | 34 | 39.09 |
|  | 3 | 72 | 40.17 |
|  | 2 | 53 | 41.63 |
|  | 4 | 29 | 49.48 |
|  | significance |  | .103 |

| **MMSE** | | | | | | |
| --- | --- | --- | --- | --- | --- | --- |
|  | Severity | N | alpha = 0.05 subset | | | |
|  |  |  | 1 | 2 | 3 | 4 |
| Student-Newman-Keuls comparation^a,b^ | 4 | 29 | .97 |  |  |  |
|  | 3 | 72 |  | 5.15 |  |  |
|  | 2 | 53 |  |  | 17.74 |  |
|  | 1 | 34 |  |  |  | 22.79 |
|  | significance |  | 1.000 | 1.000 | 1.000 | 1.000 |

| **GCS** | | | | | |
| --- | --- | --- | --- | --- | --- |
|  | Severity | N | alpha = 0.05 subset | | |
|  |  |  | 1 | 2 | 3 |
| Student-Newman-Keuls comparation^a,b^ | 4 | 29 | 4.03 |  |  |
|  | 3 | 72 |  | 9.03 |  |
|  | 2 | 53 |  |  | 14.32 |
|  | 1 | 34 |  |  | 14.94 |
|  | significance |  | 1.000 | 1.000 | .245 |
|  | | | | | |
|  | | | | | |
|  | | | | | |
