# Supplementary material for: Serum Total Cholinesterase Activity on Admission Is Associated with Disease Severity and Outcome in Patients with Traumatic Brain Injury
Source: PLoS One. 2015 Jun 24;10(6):e0129082. doi: 10.1371/journal.pone.0129082 (PMC4479571; doi:10.1371/journal.pone.0129082)
Supplement: S4 File — (DOCX) [file pone.0129082.s005.docx]

| **Total population** | | | | | | | | | | | | | | | | |
| --- | --- | --- | --- | --- | --- | --- | --- | --- | --- | --- | --- | --- | --- | --- | --- | --- |
|  | | ChE | WBC | Lymphocyte | neutrophil | Lymph% | monocyte | Hospital LOS | ICU LOS | APACHE II | age | outcome | sepsis | GCS | MMSE | Severity |
| N | valid | 173 | 185 | 183 | 181 | 184 | 183 | 187 | 188 | 187 | 188 | 188 | 188 | 188 | 188 | 188 |
|  | omit | 32 | 20 | 22 | 24 | 21 | 22 | 18 | 17 | 18 | 17 | 17 | 17 | 17 | 17 | 17 |
| MEAN | | 6.8530 | 12.42 | 1.44 | 10.15 | 13.64 | .67 | 32.08 | 21.06 | 14.02 | 41.82 | .22 | .38 | 10.82 | 11.24 | 2.51 |
| SEM | | .16801 | .430 | .099 | .516 | .820 | .028 | 6.848 | 5.830 | .557 | 1.512 | .030 | .036 | .332 | .866 | .070 |
| SD | | 2.20979 | 5.848 | 1.335 | 6.947 | 11.129 | .380 | 93.648 | 79.935 | 7.616 | 20.732 | .418 | .487 | 4.549 | 11.880 | .962 |
| Variation | | 4.883 | 34.193 | 1.781 | 48.256 | 123.849 | .144 | 8770.010 | 6389.680 | 58.000 | 429.818 | .174 | .238 | 20.694 | 141.127 | .925 |
| percentiles | 25 | 5.3850 | 8.35 | .70 | 5.90 | 6.00 | .40 | 8.00 | 2.00 | 8.00 | 25.00 | .00 | .00 | 7.00 | .00 | 2.00 |
|  | 50 | 6.7900 | 11.20 | 1.20 | 9.20 | 9.55 | .60 | 13.00 | 5.00 | 13.00 | 42.50 | .00 | .00 | 13.00 | .00 | 3.00 |
|  | 75 | 8.0000 | 15.45 | 1.90 | 13.30 | 18.93 | .80 | 24.00 | 10.00 | 19.00 | 56.00 | .00 | 1.00 | 15.00 | 24.00 | 3.00 |

| **Septic patients statistic analysis** | | | | | | | | | | | | | | | | |
| --- | --- | --- | --- | --- | --- | --- | --- | --- | --- | --- | --- | --- | --- | --- | --- | --- |
|  | | type | age | ChE | WBC | Lymphcyte | Neutrophil | lymphoper | monocyte | HospitalLOS | ICULOS | APACHE | Survival | sepsis | GCS | MMSE |
| N | valid | 72 | 72 | 59 | 70 | 70 | 69 | 70 | 69 | 71 | 72 | 71 | 72 | 72 | 72 | 72 |
|  | omit | 0 | 0 | 13 | 2 | 2 | 3 | 2 | 3 | 1 | 0 | 1 | 0 | 0 | 0 | 0 |
| MEAN | | 3.3611 | 43.3611 | 6.1142 | 15.4314 | 1.4564 | 12.2635 | 9.7614 | .7064 | 52.5775 | 41.4861 | 20.3662 | .5278 | 1.0000 | 6.4444 | 2.0139 |
| SEM | | .06350 | 2.22232 | .24793 | .81124 | .21666 | .70769 | 1.04685 | .04907 | 16.86454 | 14.41174 | .82741 | .05925 | .00000 | .40772 | .73198 |
| Median | | 3.0000 | 43.0000 | 5.8000 | 14.9850 | 1.0650 | 11.9000 | 7.2500 | .6200 | 17.0000 | 9.0000 | 20.0000 | 1.0000 | 1.0000 | 6.0000 | .0000 |
| Mode | | 3.00 | 25.00 | 4.80^a^ | 14.30 | .50 | 13.00^a^ | 2.90^a^ | .40 | 4.00^a^ | 4.00^a^ | 13.00 | 1.00 | 1.00 | 3.00 | .00 |
| SD | | .53879 | 18.85699 | 1.90435 | 6.78736 | 1.81267 | 5.87850 | 8.75860 | .40759 | 142.10315 | 122.28770 | 6.97186 | .50273 | .00000 | 3.45958 | 6.21106 |
| Variation | | .290 | 355.586 | 3.627 | 46.068 | 3.286 | 34.557 | 76.713 | .166 | 20193.305 | 14954.281 | 48.607 | .253 | .000 | 11.969 | 38.577 |
| skewness | | .026 | .686 | .210 | 1.651 | 5.534 | 1.601 | 2.349 | .902 | 5.916 | 4.528 | .451 | -.114 |  | 1.030 | 3.070 |
| Skewness SEM | | .283 | .283 | .311 | .287 | .287 | .289 | .287 | .289 | .285 | .283 | .285 | .283 | .283 | .283 | .283 |
| peak | | -.904 | -.327 | -.721 | 4.721 | 38.235 | 6.423 | 6.768 | .737 | 38.968 | 21.563 | -.491 | -2.045 |  | .203 | 8.274 |
| [kurtosis](javascript:void(0);) SEM | | .559 | .559 | .613 | .566 | .566 | .570 | .566 | .570 | .563 | .559 | .563 | .559 | .559 | .559 | .559 |
| range | | 2.00 | 75.00 | 7.79 | 40.07 | 14.20 | 39.30 | 44.80 | 2.00 | 1064.00 | 768.00 | 29.00 | 1.00 | .00 | 12.00 | 28.00 |
| percentiles | 25 | 3.0000 | 26.2500 | 4.8000 | 11.0525 | .5825 | 8.5250 | 3.9750 | .4000 | 7.0000 | 5.0000 | 15.0000 | .0000 | 1.0000 | 3.2500 | .0000 |
|  | 50 | 3.0000 | 43.0000 | 5.8000 | 14.9850 | 1.0650 | 11.9000 | 7.2500 | .6200 | 17.0000 | 9.0000 | 20.0000 | 1.0000 | 1.0000 | 6.0000 | .0000 |
|  | 75 | 4.0000 | 53.7500 | 7.5000 | 17.8250 | 1.7050 | 14.9500 | 13.0250 | .9500 | 38.0000 | 16.0000 | 25.0000 | 1.0000 | 1.0000 | 8.0000 | .0000 |
|  | | | | | | | | | | | | | | | | |

| **Non-infectious Septic patients statistic analysis** | | | | | | | | | | | | | | | | |
| --- | --- | --- | --- | --- | --- | --- | --- | --- | --- | --- | --- | --- | --- | --- | --- | --- |
|  | | type | age | ChE | WBC | Lymphcyte | Neutrophil | lymphoper | monocyte | HospitalLOS | ICULOS | APACHE | Survival | sepsis | GCS | MMSE |
| N | Valid | 116 | 116 | 114 | 115 | 113 | 112 | 114 | 114 | 116 | 116 | 116 | 116 | 116 | 116 | 116 |
|  | omit | 0 | 0 | 2 | 1 | 3 | 4 | 2 | 2 | 0 | 0 | 0 | 0 | 0 | 0 | 0 |
| MEAN | | 1.9828 | 40.8672 | 7.2354 | 10.5943 | 1.4379 | 8.8540 | 16.0167 | .6467 | 19.5345 | 8.3793 | 10.1379 | .0345 | .0000 | 13.5345 | 16.9741 |
| SEM | | .07138 | 2.02779 | .21232 | .39973 | .08773 | .68539 | 1.10306 | .03391 | 3.56565 | 2.51567 | .45632 | .01702 | .00000 | .24287 | 1.01373 |
| Median | | 2.0000 | 42.0000 | 7.1350 | 9.6800 | 1.2000 | 7.6000 | 12.6500 | .6000 | 12.0000 | 4.0000 | 9.0000 | .0000 | .0000 | 15.0000 | 22.0000 |
| Mode | | 2.00 | 46.00 | 7.40 | 8.50^a^ | .60 | 5.90 | 6.00^a^ | .50 | 12.00 | .00 | 6.00 | .00 | .00 | 15.00 | .00 |
| SD | | .76877 | 21.83995 | 2.26692 | 4.28657 | .93255 | 7.25344 | 11.77748 | .36201 | 38.40322 | 27.09454 | 4.91475 | .18326 | .00000 | 2.61578 | 10.91824 |
| Variation | | .591 | 476.984 | 5.139 | 18.375 | .870 | 52.612 | 138.709 | .131 | 1474.807 | 734.114 | 24.155 | .034 | .000 | 6.842 | 119.208 |
| skewness | | .146 | .187 | 2.281 | 1.029 | 2.258 | 5.563 | 1.450 | 2.029 | 8.177 | 8.116 | 1.049 | 5.170 |  | -1.963 | -.752 |
| Skewness SEM | | .225 | .225 | .226 | .226 | .227 | .228 | .226 | .226 | .225 | .225 | .225 | .225 | .225 | .225 | .225 |
| peak | | -.971 | -.478 | 10.114 | 1.280 | 7.512 | 45.309 | 2.545 | 8.328 | 75.780 | 72.469 | .939 | 25.158 |  | 3.161 | -1.165 |
| [kurtosis](javascript:void(0);) SEM | | .446 | .446 | .449 | .447 | .451 | .453 | .449 | .449 | .446 | .446 | .446 | .446 | .446 | .446 | .446 |
| range | | 3.00 | 94.40 | 15.55 | 24.96 | 5.80 | 70.00 | 60.80 | 2.56 | 386.00 | 264.00 | 23.00 | 1.00 | .00 | 12.00 | 28.00 |
| percentiles | 25 | 1.0000 | 23.2500 | 5.7450 | 7.7000 | .8000 | 5.1250 | 7.1000 | .4000 | 8.0000 | .0000 | 6.0000 | .0000 | .0000 | 13.0000 | .0000 |
|  | 50 | 2.0000 | 42.0000 | 7.1350 | 9.6800 | 1.2000 | 7.6000 | 12.6500 | .6000 | 12.0000 | 4.0000 | 9.0000 | .0000 | .0000 | 15.0000 | 22.0000 |
|  | 75 | 3.0000 | 56.7500 | 8.1475 | 12.9000 | 1.9000 | 10.9500 | 23.6000 | .8000 | 20.7500 | 8.0000 | 13.0000 | .0000 | .0000 | 15.0000 | 25.0000 |
|  | | | | | | | | | | | | | | | | |
