# Supplementary material for: Serum Total Cholinesterase Activity on Admission Is Associated with Disease Severity and Outcome in Patients with Traumatic Brain Injury
Source: PLoS One. 2015 Jun 24;10(6):e0129082. doi: 10.1371/journal.pone.0129082 (PMC4479571; doi:10.1371/journal.pone.0129082)
Supplement: S5 File — (DOCX) [file pone.0129082.s006.docx]

| **Survival patients statistic analysis** | | | | | | | | | | | | | |
| --- | --- | --- | --- | --- | --- | --- | --- | --- | --- | --- | --- | --- | --- |
|  | | age | ChE | WBC | Lym | Neutr | Lymper | monocyte | HospitalLOS | ICULOS | APACHE | GCS | MMSE |
| N | valid | 147 | 143 | 144 | 144 | 142 | 145 | 145 | 146 | 147 | 146 | 147 | 147 |
|  | omit | 0 | 4 | 3 | 3 | 5 | 2 | 2 | 1 | 0 | 1 | 0 | 0 |
| MEAN | | 39.85 | 7.0129 | 11.28 | 1.42 | 9.40 | 14.21 | .68 | 36.14 | 18.70 | 11.34 | 12.33 | 14.00 |
| SEM | | 1.677 | .18251 | .373 | .117 | .567 | .938 | .031 | 8.695 | 5.399 | .448 | .308 | .966 |
| Medianomit | | 41.00 | 7.0000 | 10.10 | 1.15 | 8.40 | 9.60 | .60 | 14.00 | 4.00 | 11.00 | 15.00 | 20.00 |
| SD | | 20.338 | 2.18246 | 4.478 | 1.402 | 6.761 | 11.298 | .375 | 105.059 | 65.464 | 5.417 | 3.736 | 11.708 |
| variation | | 413.616 | 4.763 | 20.054 | 1.967 | 45.705 | 127.646 | .141 | 11037.421 | 4285.470 | 29.344 | 13.961 | 137.068 |
| range | | 94 | 16.09 | 25 | 14 | 70 | 61 | 3 | 1063 | 528 | 23 | 12 | 28 |
| Min | | 1 | 3.16 | 2 | 0 | 0 | 2 | 0 | 2 | 0 | 4 | 3 | 0 |
| Max | | 95 | 19.25 | 27 | 14 | 70 | 63 | 3 | 1065 | 528 | 27 | 15 | 28 |
| Percentile | 25 | 24.00 | 5.5000 | 8.00 | .70 | 5.75 | 6.15 | .40 | 9.00 | 2.00 | 6.00 | 10.00 | .00 |
|  | 50 | 41.00 | 7.0000 | 10.10 | 1.15 | 8.40 | 9.60 | .60 | 14.00 | 4.00 | 11.00 | 15.00 | 20.00 |
|  | 75 | 54.00 | 8.0000 | 14.28 | 1.80 | 12.61 | 20.85 | .80 | 25.25 | 10.00 | 15.00 | 15.00 | 25.00 |

| **Non-survival patients statistic analysis** | | | | | | | | | | | | | |
| --- | --- | --- | --- | --- | --- | --- | --- | --- | --- | --- | --- | --- | --- |
|  | | age | ChE | WBC | lym | neutr | lymper | monocyte | hospitalLOS | ICULOS | APACHE | MMSE | GCS |
| N | valid | 42 | 31 | 42 | 40 | 40 | 40 | 39 | 42 | 42 | 42 | 42 | 42 |
|  | omit | 0 | 11 | 0 | 2 | 2 | 2 | 3 | 0 | 0 | 0 | 0 | 0 |
| MEAN | | 49.83 | 6.0139 | 16.27 | 1.51 | 12.75 | 11.4550 | .61 | 17.48 | 28.90 | 23.36 | 1.83 | 5.64 |
| SEM | | 3.309 | .39893 | 1.235 | .166 | 1.103 | 1.61490 | .064 | 3.394 | 18.131 | 1.030 | .975 | .476 |
| 中位數 | | 47.00 | 5.7000 | 15.47 | 1.25 | 11.92 | 9.1000 | .50 | 9.00 | 6.50 | 22.50 | .00 | 5.00 |
| SD | | 21.443 | 2.22113 | 8.004 | 1.050 | 6.976 | 10.21354 | .397 | 21.993 | 117.502 | 6.673 | 6.320 | 3.083 |
| Variate | | 459.801 | 4.933 | 64.061 | 1.103 | 48.663 | 104.316 | .157 | 483.670 | 13806.820 | 44.528 | 39.947 | 9.503 |
| Min | | 17 | 2.01 | 2 | 0 | 0 | .90 | 0 | 1 | 0 | 13 | 0 | 3 |
| Max | | 93 | 9.80 | 42 | 4 | 39 | 45.70 | 2 | 90 | 768 | 38 | 28 | 15 |
| Percentile | 25 | 32.00 | 4.2700 | 10.54 | .65 | 8.70 | 4.1250 | .32 | 5.00 | 4.00 | 18.00 | .00 | 3.00 |
|  | 50 | 47.00 | 5.7000 | 15.47 | 1.25 | 11.92 | 9.1000 | .50 | 9.00 | 6.50 | 22.50 | .00 | 5.00 |
|  | 75 | 71.25 | 7.8000 | 18.67 | 2.05 | 15.70 | 13.3750 | .80 | 17.50 | 11.75 | 29.00 | .00 | 7.25 |
