# Supplementary material for: Serum Total Cholinesterase Activity on Admission Is Associated with Disease Severity and Outcome in Patients with Traumatic Brain Injury
Source: PLoS One. 2015 Jun 24;10(6):e0129082. doi: 10.1371/journal.pone.0129082 (PMC4479571; doi:10.1371/journal.pone.0129082)
Supplement: S7 File — (DOCX) [file pone.0129082.s008.docx]

| **ChE correlate with other parameters** | | | | | | | | | | | | | |
| --- | --- | --- | --- | --- | --- | --- | --- | --- | --- | --- | --- | --- | --- |
|  | | ChE | WBC | Lymph | neutrop | lymphper | monocyte | hospitalLOS | ICULOS | APACHE | GCS | MMSE | age |
| ChE | Pearson’ correlation | 1 | -.210^**^ | .106 | -.243^**^ | .262^**^ | -.128 | -.078 | -.108 | -.208^**^ | .174^*^ | .167^*^ | -.129 |
|  | Significance (2-tailed) |  | .006 | .171 | .002 | .001 | .098 | .311 | .157 | .006 | .022 | .028 | .090 |
|  | N | 173 | 171 | 169 | 167 | 170 | 168 | 172 | 173 | 172 | 173 | 173 | 173 |
| WBC | Pearson’ correlation | -.210^**^ | 1 | .071 | .546^**^ | -.355^**^ | .446^**^ | .003 | -.039 | .459^**^ | -.443^**^ | -.371^**^ | .043 |
|  | Significance (2-tailed) | .006 |  | .345 | .000 | .000 | .000 | .969 | .599 | .000 | .000 | .000 | .562 |
|  | N | 171 | 185 | 180 | 178 | 181 | 180 | 184 | 185 | 185 | 185 | 185 | 185 |
| Lymph | Pearson’ correlation | .106 | .071 | 1 | -.076 | .439^**^ | .300^**^ | -.059 | -.018 | -.087 | .014 | -.069 | -.234^**^ |
|  | Significance (2-tailed) | .171 | .345 |  | .313 | .000 | .000 | .429 | .806 | .242 | .853 | .351 | .001 |
|  | N | 169 | 180 | 183 | 178 | 183 | 181 | 182 | 183 | 182 | 183 | 183 | 183 |
| neutrop | Pearson’ correlation | -.243^**^ | .546^**^ | -.076 | 1 | -.318^**^ | .190^*^ | .006 | .001 | .306^**^ | -.258^**^ | -.242^**^ | .147^*^ |
|  | Significance (2-tailed) | .002 | .000 | .313 |  | .000 | .011 | .933 | .988 | .000 | .000 | .001 | .048 |
|  | N | 167 | 178 | 178 | 181 | 179 | 179 | 180 | 181 | 180 | 181 | 181 | 181 |
| lymphper | Pearson’ correlation | .262^**^ | -.355^**^ | .439^**^ | -.318^**^ | 1 | -.015 | -.063 | .007 | -.328^**^ | .295^**^ | .158^*^ | -.298^**^ |
|  | Significance (2-tailed) | .001 | .000 | .000 | .000 |  | .837 | .395 | .925 | .000 | .000 | .032 | .000 |
|  | N | 170 | 181 | 183 | 179 | 184 | 182 | 183 | 184 | 183 | 184 | 184 | 184 |
| monocyte | Pearson’ correlation | -.128 | .446^**^ | .300^**^ | .190^*^ | -.015 | 1 | .006 | .060 | .085 | -.081 | -.225^**^ | -.134 |
|  | Significance (2-tailed) | .098 | .000 | .000 | .011 | .837 |  | .935 | .418 | .256 | .277 | .002 | .071 |
|  | N | 168 | 180 | 181 | 179 | 182 | 183 | 182 | 183 | 182 | 183 | 183 | 183 |
| hospitalLOS | Pearson’ correlation | -.078 | .003 | -.059 | .006 | -.063 | .006 | 1 | .165^*^ | .175^*^ | -.207^**^ | -.163^*^ | -.061 |
|  | Significance (2-tailed) | .311 | .969 | .429 | .933 | .395 | .935 |  | .024 | .017 | .005 | .025 | .408 |
|  | N | 172 | 184 | 182 | 180 | 183 | 182 | 187 | 187 | 186 | 187 | 187 | 187 |
| ICULOS | Pearson’ correlation | -.108 | -.039 | -.018 | .001 | .007 | .060 | .165^*^ | 1 | .059 | -.012 | -.116 | .039 |
|  | Significance (2-tailed) | .157 | .599 | .806 | .988 | .925 | .418 | .024 |  | .423 | .874 | .111 | .594 |
|  | N | 173 | 185 | 183 | 181 | 184 | 183 | 187 | 188 | 187 | 188 | 188 | 188 |
| APACHE | Pearson’ correlation | -.208^**^ | .459^**^ | -.087 | .306^**^ | -.328^**^ | .085 | .175^*^ | .059 | 1 | -.811^**^ | -.618^**^ | .400^**^ |
|  | Significance (2-tailed) | .006 | .000 | .242 | .000 | .000 | .256 | .017 | .423 |  | .000 | .000 | .000 |
|  | N | 172 | 185 | 182 | 180 | 183 | 182 | 186 | 187 | 187 | 187 | 187 | 187 |
| GCS | Pearson’ correlation | .174^*^ | -.443^**^ | .014 | -.258^**^ | .295^**^ | -.081 | -.207^**^ | -.012 | -.811^**^ | 1 | .740^**^ | -.075 |
|  | Significance (2-tailed) | .022 | .000 | .853 | .000 | .000 | .277 | .005 | .874 | .000 |  | .000 | .309 |
|  | N | 173 | 185 | 183 | 181 | 184 | 183 | 187 | 188 | 187 | 188 | 188 | 188 |
| MMSE | Pearson’ correlation | .167^*^ | -.371^**^ | -.069 | -.242^**^ | .158^*^ | -.225^**^ | -.163^*^ | -.116 | -.618^**^ | .740^**^ | 1 | .015 |
|  | Significance (2-tailed) | .028 | .000 | .351 | .001 | .032 | .002 | .025 | .111 | .000 | .000 |  | .834 |
|  | N | 173 | 185 | 183 | 181 | 184 | 183 | 187 | 188 | 187 | 188 | 188 | 188 |
| age | Pearson’ correlation | -.129 | .043 | -.234^**^ | .147^*^ | -.298^**^ | -.134 | -.061 | .039 | .400^**^ | -.075 | .015 | 1 |
|  | Significance (2-tailed) | .090 | .562 | .001 | .048 | .000 | .071 | .408 | .594 | .000 | .309 | .834 |  |
|  | N | 173 | 185 | 183 | 181 | 184 | 183 | 187 | 188 | 187 | 188 | 188 | 188 |
| **. P= 0.01, *. P= 0.05 | | | | | | | | | | | | | |
|  | | | | | | | | | | | | | |

**Non-parametric correlation**

| **Correlation** | | | | | | | | | | | | | | |
| --- | --- | --- | --- | --- | --- | --- | --- | --- | --- | --- | --- | --- | --- | --- |
|  | | | ChE | WBC | Lymph | neutrop | lymphper | monocyte | hospitalLOS | ICULOS | APACHE | GCS | MMSE | age |
| Spearman’ rho | ChE | Coefficient | 1.000 | -.154^*^ | .208^**^ | -.184^*^ | .245^**^ | -.181^*^ | -.141 | -.187^*^ | -.210^**^ | .193^*^ | .151^*^ | -.071 |
|  |  | significance (2-tailed) | . | .044 | .007 | .018 | .001 | .019 | .066 | .014 | .006 | .011 | .048 | .352 |
|  |  | N | 173 | 171 | 169 | 167 | 170 | 168 | 172 | 173 | 172 | 173 | 173 | 173 |
|  | WBC | Coefficient | -.154^*^ | 1.000 | -.105 | .739^**^ | -.531^**^ | .368^**^ | .057 | .172^*^ | .445^**^ | -.488^**^ | -.392^**^ | .060 |
|  |  | significance (2-tailed) | .044 | . | .159 | .000 | .000 | .000 | .438 | .019 | .000 | .000 | .000 | .414 |
|  |  | N | 171 | 185 | 180 | 178 | 181 | 180 | 184 | 185 | 185 | 185 | 185 | 185 |
|  | Lymph | Coefficient | .208^**^ | -.105 | 1.000 | -.277^**^ | .798^**^ | .257^**^ | -.200^**^ | -.197^**^ | -.192^**^ | .214^**^ | .088 | -.217^**^ |
|  |  | significance (2-tailed) | .007 | .159 | . | .000 | .000 | .000 | .007 | .007 | .009 | .004 | .238 | .003 |
|  |  | N | 169 | 180 | 183 | 178 | 183 | 181 | 182 | 183 | 182 | 183 | 183 | 183 |
|  | neutrop | Coefficient | -.184^*^ | .739^**^ | -.277^**^ | 1.000 | -.525^**^ | .166^*^ | .123 | .204^**^ | .406^**^ | -.432^**^ | -.341^**^ | .090 |
|  |  | significance (2-tailed) | .018 | .000 | .000 | . | .000 | .026 | .100 | .006 | .000 | .000 | .000 | .226 |
|  |  | N | 167 | 178 | 178 | 181 | 179 | 179 | 180 | 181 | 180 | 181 | 181 | 181 |
|  | lymphper | Coefficient | .245^**^ | -.531^**^ | .798^**^ | -.525^**^ | 1.000 | .002 | -.218^**^ | -.257^**^ | -.380^**^ | .407^**^ | .294^**^ | -.205^**^ |
|  |  | significance (2-tailed) | .001 | .000 | .000 | .000 | . | .975 | .003 | .000 | .000 | .000 | .000 | .005 |
|  |  | N | 170 | 181 | 183 | 179 | 184 | 182 | 183 | 184 | 183 | 184 | 184 | 184 |
|  | monocyte | Coefficient | -.181^*^ | .368^**^ | .257^**^ | .166^*^ | .002 | 1.000 | .143 | -.016 | .089 | -.048 | -.226^**^ | -.081 |
|  |  | significance (2-tailed) | .019 | .000 | .000 | .026 | .975 | . | .055 | .835 | .234 | .519 | .002 | .278 |
|  |  | N | 168 | 180 | 181 | 179 | 182 | 183 | 182 | 183 | 182 | 183 | 183 | 183 |
|  | hospitalLOS | Coefficient | -.141 | .057 | -.200^**^ | .123 | -.218^**^ | .143 | 1.000 | .432^**^ | .129 | -.169^*^ | -.253^**^ | -.048 |
|  |  | significance (2-tailed) | .066 | .438 | .007 | .100 | .003 | .055 | . | .000 | .080 | .021 | .000 | .512 |
|  |  | N | 172 | 184 | 182 | 180 | 183 | 182 | 187 | 187 | 186 | 187 | 187 | 187 |
|  | ICULOS | Coefficient | -.187^*^ | .172^*^ | -.197^**^ | .204^**^ | -.257^**^ | -.016 | .432^**^ | 1.000 | .349^**^ | -.449^**^ | -.374^**^ | .064 |
|  |  | significance (2-tailed) | .014 | .019 | .007 | .006 | .000 | .835 | .000 | . | .000 | .000 | .000 | .384 |
|  |  | N | 173 | 185 | 183 | 181 | 184 | 183 | 187 | 188 | 187 | 188 | 188 | 188 |
|  | APACHE | Coefficient | -.210^**^ | .445^**^ | -.192^**^ | .406^**^ | -.380^**^ | .089 | .129 | .349^**^ | 1.000 | -.803^**^ | -.643^**^ | .390^**^ |
|  |  | significance (2-tailed) | .006 | .000 | .009 | .000 | .000 | .234 | .080 | .000 | . | .000 | .000 | .000 |
|  |  | N | 172 | 185 | 182 | 180 | 183 | 182 | 186 | 187 | 187 | 187 | 187 | 187 |
|  | GCS | Coefficient | .193^*^ | -.488^**^ | .214^**^ | -.432^**^ | .407^**^ | -.048 | -.169^*^ | -.449^**^ | -.803^**^ | 1.000 | .734^**^ | -.093 |
|  |  | significance (2-tailed) | .011 | .000 | .004 | .000 | .000 | .519 | .021 | .000 | .000 | . | .000 | .206 |
|  |  | N | 173 | 185 | 183 | 181 | 184 | 183 | 187 | 188 | 187 | 188 | 188 | 188 |
|  | MMSE | Coefficient | .151^*^ | -.392^**^ | .088 | -.341^**^ | .294^**^ | -.226^**^ | -.253^**^ | -.374^**^ | -.643^**^ | .734^**^ | 1.000 | .017 |
|  |  | significance (2-tailed) | .048 | .000 | .238 | .000 | .000 | .002 | .000 | .000 | .000 | .000 | . | .820 |
|  |  | N | 173 | 185 | 183 | 181 | 184 | 183 | 187 | 188 | 187 | 188 | 188 | 188 |
|  | age | Coefficient | -.071 | .060 | -.217^**^ | .090 | -.205^**^ | -.081 | -.048 | .064 | .390^**^ | -.093 | .017 | 1.000 |
|  |  | significance (2-tailed) | .352 | .414 | .003 | .226 | .005 | .278 | .512 | .384 | .000 | .206 | .820 | . |
|  |  | N | 173 | 185 | 183 | 181 | 184 | 183 | 187 | 188 | 187 | 188 | 188 | 188 |
| **. P= 0.01 | | | | | | | | | | | | | | |
| **. P= 0.01 | | | | | | | | | | | | | | |
