# Supplementary material for: Serum Total Cholinesterase Activity on Admission Is Associated with Disease Severity and Outcome in Patients with Traumatic Brain Injury
Source: PLoS One. 2015 Jun 24;10(6):e0129082. doi: 10.1371/journal.pone.0129082 (PMC4479571; doi:10.1371/journal.pone.0129082)
Supplement: S9 File — (DOCX) [file pone.0129082.s010.docx]

**Cox Regression**

| **Omnibus Tests of Model Coefficients^a,b^** | | | | | | | | | | | | | | | | | | |
| --- | --- | --- | --- | --- | --- | --- | --- | --- | --- | --- | --- | --- | --- | --- | --- | --- | --- | --- |
| -2 Log Likelihood | | Overall (score) | | | | | | Change From Previous Step | | | | | | Change From Previous Block | | | | |
|  |  | Chi-square | | df | | Sig. | | Chi-square | | df | | Sig. | | Chi-square | | df | | Sig. |
| 1426.357 | | 13.226 | | 6 | | .040 | | 12.380 | | 6 | | .054 | | 12.380 | | 6 | | .054 |
| a. Beginning Block Number 0, initial Log Likelihood function: -2 Log likelihood: 1438.737 | | | | | | | | | | | | | | | |  | |  |
| b. Beginning Block Number 1. Method = Enter | | | | | | | |  | |  | |  | |  | |  | |  |
| **Variables in the Equation** | | | | | | | | | | | | | | | | |  |  |
|  | B | | SE | | Wald | | df | | Sig. | | Exp(B) | | 95.0% CI for Exp(B) | | | |  |  |
|  |  |  |  |  |  |  |  |  |  |  |  |  | Lower | | Upper | |  |  |
| Age | .006 | | .004 | | 1.902 | | 1 | | .168 | | 1.006 | | .998 | | 1.014 | |  |  |
| Lymphocyte | .216 | | .107 | | 4.050 | | 1 | | .044 | | 1.241 | | 1.006 | | 1.531 | |  |  |
| Neutrophil | -.003 | | .015 | | .029 | | 1 | | .865 | | .997 | | .969 | | 1.027 | |  |  |
| monocyte | -.774 | | .296 | | 6.813 | | 1 | | .009 | | .461 | | .258 | | .825 | |  |  |
| WBC | .059 | | .022 | | 7.253 | | 1 | | .007 | | 1.061 | | 1.016 | | 1.107 | |  |  |
| ChE | -.004 | | .045 | | .007 | | 1 | | .934 | | .996 | | .912 | | 1.089 | |  |  |
